# Converging Robotic Technologies in Targeted Neural Rehabilitation: A Review of Emerging Solutions and Challenges

**DOI:** 10.3390/s21062084

**Published:** 2021-03-16

**Authors:** Kostas Nizamis, Alkinoos Athanasiou, Sofia Almpani, Christos Dimitrousis, Alexander Astaras

**Affiliations:** 1 Department of Design, Production and Management, University of Twente, 7522 NB Enschede, The Netherlands; 2 Lab of Medical Physics, School of Medicine, Faculty of Health Sciences, Aristotle University of Thessaloniki, 54124 Thessaloniki, Greece; athalkinoos@auth.gr (A.A.); astaras@act.edu (A.A.); 3 School of Electrical and Computer Engineering, National Technical University of Athens, 15773 Athens, Greece; salmpani@mail.ntua.gr; 4 Department of Computer Science, American College of Thessaloniki, 55535 Thessaloniki, Greece; c.dimitrousis@gmail.com

**Keywords:** artificial intelligence, brain–computer interfaces, exoskeleton, human–robot interaction, neurological disability, neurorehabilitation, robotics, neural interfaces

## Abstract

Recent advances in the field of neural rehabilitation, facilitated through technological innovation and improved neurophysiological knowledge of impaired motor control, have opened up new research directions. Such advances increase the relevance of existing interventions, as well as allow novel methodologies and technological synergies. New approaches attempt to partially overcome long-term disability caused by spinal cord injury, using either invasive bridging technologies or noninvasive human–machine interfaces. Muscular dystrophies benefit from electromyography and novel sensors that shed light on underlying neuromotor mechanisms in people with Duchenne. Novel wearable robotics devices are being tailored to specific patient populations, such as traumatic brain injury, stroke, and amputated individuals. In addition, developments in robot-assisted rehabilitation may enhance motor learning and generate movement repetitions by decoding the brain activity of patients during therapy. This is further facilitated by artificial intelligence algorithms coupled with faster electronics. The practical impact of integrating such technologies with neural rehabilitation treatment can be substantial. They can potentially empower nontechnically trained individuals—namely, family members and professional carers—to alter the programming of neural rehabilitation robotic setups, to actively get involved and intervene promptly at the point of care. This narrative review considers existing and emerging neural rehabilitation technologies through the perspective of replacing or restoring functions, enhancing, or improving natural neural output, as well as promoting or recruiting dormant neuroplasticity. Upon conclusion, we discuss the future directions for neural rehabilitation research, diagnosis, and treatment based on the discussed technologies and their major roadblocks. This future may eventually become possible through technological evolution and convergence of mutually beneficial technologies to create hybrid solutions.

## 1. Introduction

Neural rehabilitation, to the extent this is possible, aims to restore the functionality of impaired neurological circuits or complement remaining functionality. This aims to enhance patient independence and quality of life through exploitation of neural plasticity [1,2]. Predominant focus of the neural rehabilitation field lies on restoration of sensorimotor control and functions. Neural plasticity is based on the hypothesis that central nervous system (CNS) and peripheral nervous system (PNS) circuits can be retrained after a lesion in order to facilitate effective rehabilitation [3]. The main neural rehabilitation research approaches can be described as bottom-up procedures where, by acting on the affected limbs, one aims to influence the CNS; however, the exact afferent mechanisms of neuroplasticity behind this approach are still unknown [4]. Neural prosthetic grasping hands and legs, support exoskeletons, and body-weight support/robotic treadmill systems all share the bottom-up approach, where plasticity is driven by the device and by rehabilitation practice. Recently an emerging research trend is exploring top-down approaches as a new paradigm for exploiting neuroplasticity by first studying and modulating the state of the brain [4]. As such, rehabilitation is driven by said neuroplastic changes [4]. Brain–computer interfaces and virtual reality variants all fall in this category where neural plasticity is recruited to accommodate lasting rehabilitation effects. Additionally, there is a current focus on repetitive and intensive training; however, other principles of motor learning (such as transferability of learning to daily activities, active engagement, and problem solving) are not yet fully explored [5]. Due to its repetitive, intensive, and task-specific nature, robotics rehabilitation is a great candidate for integrating the abovementioned hypotheses for retraining the CNS [4,5].

The idea of robotic devices for rehabilitation dates back to the beginning of the previous century [1], and it is up to this day rapidly expanding [6]. Not all relevant technologies have evolved though at the same pace. Compared to wearable exoskeletons, the field of neuroprosthetics has experienced significant longstanding technological advances that led up to this point to larger clinical and market applications [7,8,9]. Such devices can facilitate neuroplasticity via targeted and repetitive exercises for the lower [2] and upper limbs [10]. Those technologies display an array of advantages. Robotic rehabilitation can reduce the burden of therapists by automating tedious and labor-intensive therapy [2] and by adapting to the specific needs of the targeted individuals [1]. Additionally, it can offer a multisensory rehabilitation experience [11] when combined with other technologies such as virtual and augmented reality (VR/AR) and gaming [12] or haptics [13]. This way it can provide additional sensory feedback to facilitate neuroplasticity and become more intuitive for individuals with cognitive deficiencies [13]. Robotic technologies have demonstrated a clear potential for rehabilitation and daily use. However, the results of robotic rehabilitation as a standalone intervention are limited in terms of clinical and functional daily life outcomes [10,14,15]. This may be attributed to problems regarding robotic control interfaces [7,10], weak or unexplored synergies between robotics and other emerging technologies, or even poor understanding of human motor control impairment [1,2]. We further expand upon these advantages and disadvantages in the relevant subsections of each technology.

Better understanding of the neurophysiological specifics that underlie impaired motor control should therefore be considered particularly important and should also drive technological rehabilitation developments to increase their clinical impact [1]. In Section 2, we discuss the application of robotic neurorehabilitation to a number of indicative impairments. These key conditions limit human motor control and present researchers with a broad range of open-ended problems capable of illustrating the aims and current clinical challenges for robotic neurorehabilitation. They include stroke (acquired CNS neuron loss of vascular cause), traumatic brain and spinal cord injury (acquired CNS injuries), and amputation (acquired PNS injury and loss of tissue) (See Section 2.1, Section 2.2, Section 2.3 and Section 2.4), as well as neuromuscular disorders such as Duchenne muscular dystrophy (DMD, see Section 2.5). Additionally, we discuss applications in mental disorders, as they may impair motor control through lack of motivation (see Section 2.6). We would like to point out that this is by no means a classification of neurological diseases; rather it is an attempt to model indicative impairments according to the specific challenges they present to the field of robotic rehabilitation [16,17,18]. As such, while other conditions could be included, most of their characteristics can be modeled by a combination of the selected impairments.

The field of rehabilitation robotics is a multidisciplinary field and involves the combination of numerous technologies [6,19,20], which we discuss in Section 3. Electrodes directly link the impaired CNS and PNS with robotic control interfaces (see Section 3.1.1). Control interfaces such as digital–neural interfaces, electromyography (EMG), and brain–computer interfaces (BCIs) enable patients with impaired motor control to communicate with assistive robotic devices (see Section 3.1.2 and Section 3.1.3). Such devices can be exoskeletons or neuroprostheses, employed either for rehabilitation or daily use (see Section 3.2). VR/AR can create immersive environments for patients undergoing rehabilitation and expand the functionality of rehabilitation robotics beyond the physical world (see Section 3.3). Lastly, artificial intelligence (AI) algorithms have the capacity to bring all these technologies together and enable the meaningful integration of control interfaces and robotic devices by decoding human motor intention and enabling safe synergistic human–robot interaction (see Section 3.4).

**Figure 1:**
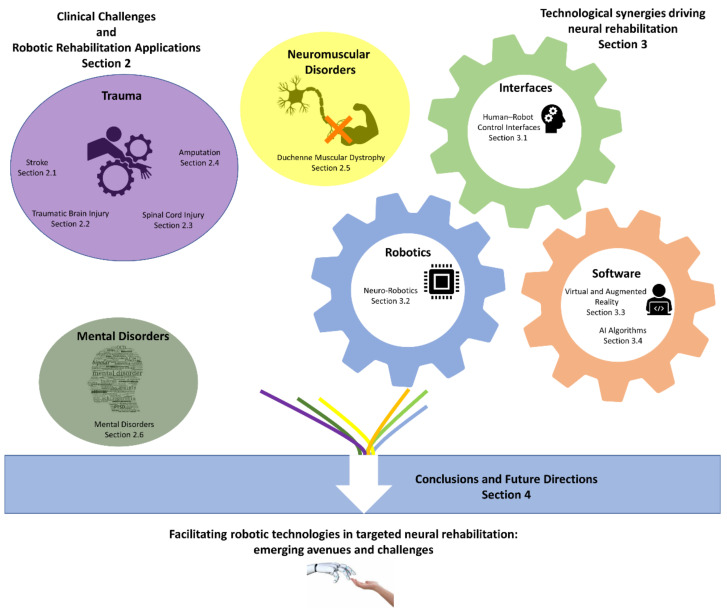
Illustrated schematic overview of the contents of this article and their connections.

As our knowledge and understanding related to impaired human motor control is improving [1,2], robotics receive great attention and funding as a rehabilitation and neuromodulation modality [7]. Additionally, several new technologies are emerging with the potential to assist in the field of neural rehabilitation [7]. Given these facts and trends, it is time to discuss how such recent advances will lead to a convergence among technological and medical insights. This discussion aims to create new research avenues for targeted robotic neural rehabilitation related to a multitude of impairments (see Section 4). Figure 1 illustrates the structure of this article.

In our research we applied a narrative review approach (see Section 5), which offers a more comprehensive way to organize and analyze the existing literature in the field of robotic neurorehabilitation compared to a systematic review. To that end, a large number of pivotal articles in peer-reviewed scientific journals were selected, which helped to identify key technologies in neurorehabilitation. In this narrative review, we provide an overview of the published literature on robotic rehabilitation combined with neuroplasticity principles and a number of key impairments that can benefit from neurorehabilitation. Our aim is to highlight recent advancements in robotic rehabilitation technology and insights on impaired motor control and to offer an integrative view on how such new knowledge from diverse fields can be combined to benefit robotic neurorehabilitation. As a second step, we are considering a new generation of robotic rehabilitation technologies, which will be implemented on a larger scale and result in better, faster, and less expensive clinical and functional rehabilitation outcomes.

## 2. Clinical Challenges and Robotic Rehabilitation Applications

### 2.1. Stroke

Stroke refers to the interruption of blood supply and drainage to the brain or to the interruption of brain blood vessel wall continuity and extravasation of blood that leads to brain tissue damage [21]. In 2017 it led to the death of 6.2 million people worldwide [21], and the predicted acceleration of the ageing population is expected to raise these numbers even further [22]. Stroke survivors often suffer from impaired motor control of their limbs [23]. Despite traditional rehabilitation efforts that try to exploit the neuroplasticity of the brain to fully or partially restore motor control [23], stroke remains the leading cause of chronic disability in the US [23].

Robotics provide a way to deliver effective rehabilitation as a standalone modality or in combination with traditional rehabilitation [7,15,24]. This is mainly due to their ability to perform repetitive rehabilitation, to adjust the intensity of rehabilitation, to make use of a standardized training environment, and to reduce the physical burden of physiotherapists [1,2]. However, the current use of robotics for stroke rehabilitation is rather limited in clinical practice probably due to their high cost and complex implementation [15]. Additionally, their clinical effectiveness is still unclear [7,14,23] and yields relatively modest results [25] as a standalone therapy. However, robotics rehabilitation combined with other therapies (functional electrical stimulation (FES), transcranial magnetic stimulation (TMS), transcranial direct current stimulation (TDCS), VR/AR, and Botox injection) shows promising results [24]. 

Rehabilitation robots together with conventional therapy in the clinic can deliver intensive training with beneficial effects, especially early after stroke [1]. Novel developments in sensors, materials, actuation, and artificial intelligence algorithms [1,7] are expected to address their current limitations. Additionally, the development of tailored human–robot interfaces should be an integral priority for further research as it currently presents one of the main limitations for further clinical integration [7].

### 2.2. Traumatic Brain Injury

Sensorimotor, cognitive, and psychosocial function impairments, either temporary or permanent, are common long-term outcomes following acute insults to the brain due to external mechanical forces. This condition and its sequelae are covered by the term traumatic brain injury (TBI) [26]. Long-term or lifetime disability and need for neurorehabilitation due to moderate or severe TBI annually affects almost 10 million people worldwide [27]. Mild TBI, characterized by alteration of consciousness level and possibly brief loss of consciousness or post-traumatic memory loss, usually results in only minor or mild cognitive and functional disorders in the long run [28,29]. Moderate or severe TBI on the other hand usually involves widespread brain damage, diffuse axonal injury, and secondary physiological/metabolic alterations and often results in severe disability in the form of sensorimotor deficits, altered consciousness states, and neurobehavioral and affect disorders [26,30,31]. TBI patients present abnormalities to their quantitative electroencephalography (EEG) profiles, varying according to the severity of their injury [32]. Neurorehabilitation interventions based on quantitative EEG-driven neurofeedback have been considered for treating mild TBI symptoms, improving quality of life and cognitive function [33,34,35]. Cognitive functions—namely, attention, inhibition, and memory—can be also the target of robotic neurorehabilitation. Versatile affective robotics for TBI patients, especially for children, have been employed to improve cognitive impairments [36]. Furthermore, social robots are also useful in driving engagement and motivation for TBI patients during sensorimotor rehabilitation protocols [37].

Rehabilitation for TBI aims at improving the quality of life and specific disorders of TBI populations and robotics have seen a particular rise in interest for sensorimotor deficits, aiming to enhance compensatory and recovery-associated neural plasticity mechanisms [16]. Robotic technologies for the rehabilitation of TBI vary from employing treadmills and exoskeletons to robotic orthoses and hybrid systems. Demonstrating adaptive neural plasticity in addition to functional improvement should be considered an important aspect of demonstrating the added value of robotic technologies to neurorehabilitation, as tools to promote neural recovery [38]. Moreover, robotic-assisted neurological assessment of motor skills may prove sensitive enough to reveal occult visuomotor and proprioception deficits, otherwise not traceable in traditional neurological examination, and effectively guide rehabilitation interventions [39]. Robotic assistance implementations, especially wearable devices such as exoskeletons, also display further advantages. Those include reproducibility of rehabilitation training and increased support during training, especially with regard to moderate and severe sensorimotor deficits [40]. Moreover, the feasibility of a hybrid approach has been demonstrated as well. Using both a passive exoskeleton and FES for actuation of a paretic arm and training of reaching movements has initial reports of high satisfaction scores [41]. Finally, the integration of VR to neural rehabilitation protocols that use robotic technologies has demonstrated the added value it can bring to rehabilitation from TBI [42]. VR improves the sense of embodiment and has been tested in the form of Cave systems (specialized room-wide projection of virtual environments) for improving balance and gait improvement after TBI [43]. Such systems have also been tested for motor skills and affective states in moderate TBI [44], as well as for enhancing neurobehavioral function during Lokomat robotic treadmill training [45]. In conclusion, a variety of assistive, robotic, and complementary neural technologies have been tested with promising results for various TBI populations. Robotic-assisted neurorehabilitation for TBI seems to already be at a more advanced stage than for other neurological disorders. What currently is perchance lacking with regard to their scalability and wide implementation can be identified as a need for standardization of neurorehabilitation protocols and interventions.

### 2.3. Spinal Cord Injury

Motor vehicle accidents and falls account for almost 8 out of 10 incidents of spinal cord injury (SCI). This condition affects predominantly young male adults, although the average age at injury has reportedly increased in recent years to 42 years in the US [46]. Life expectancy ranges from approximately 9 to 35 years for patients suffering an injury at the age of 40, up to 20 to 53 years for patients suffering an injury at the age of 20, depending on the level of injury and the extent of neurological damage. That leads to many years of disability at a decreased overall health status [47,48], increased burden on their families and social environment [49], and greatly increased private and public health care costs and living expenses [46,50]. 

Rehabilitation of SCI victims remains a significant challenge, aimed at restoring independence in activities of daily living (ADL) and walking in those patients [51]. Still, poor neurological outcomes are common with almost one-third of injuries resulting in permanent complete paraplegia or tetraplegia [46]. Robotics have nowadays been incorporated in the mainstay of rehabilitation interventions for SCI victims [52] in the form of robotic-assisted gait training, bodyweight support treadmill systems, and exoskeletons [53,54,55], especially for patients with incomplete paralysis. Anthropomorphic robotics have also been used to study sensorimotor network function and to demonstrate BCI feasibility for SCI patients. Positive neurological outcome and motivation were shown to play a role in robotic control using a BCI channel [56].

**Figure 2:**
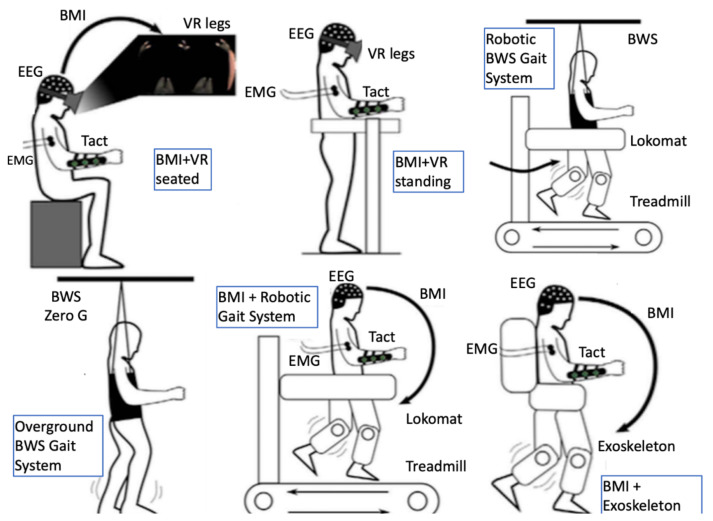
Multiple immersive man-machine interfaces and a combination of facilitating technologies have been demonstrated to have synergistic effect in promoting adaptive neuroplasticity in chronic complete spinal cord injury; figure modified from Donati et al. 2016 [58]). BMI, brain–machine interface; BWS, body weight support; EEG, electroencephalography; EMG, electromyography; Tact, tactile feedback; VR, virtual reality.

It has been demonstrated that adaptive plasticity in the sensorimotor networks of the brain and spinal cord can be promoted even in chronic stages of the condition [57]. Recently, neuroplasticity was demonstrated even in complete injuries as functional fibers crossing the injury level can be recruited into plastic changes that may lead to neurological improvement through intensive multimodal interventions [58]. The sensorimotor network is affected dynamically following an SCI [59] and research targets for promoting adaptive plasticity therein lie with different domains (for an overview see Fakhoury et al. [57]) including molecular and regenerative medicine [60,61], brain and spinal cord stimulation [62,63,64], and multimodal immersive man–machine interfaces [58,65]. The latter approach involves most of the facilitating robotics-related technologies presented later in this paper, such as BCIs, VR/AR, exoskeletons, and EMG-based assistive orthoses (Figure 2). This multimodal approach combines the advantages of maximizing immersiveness and minimizing invasiveness while currently arguably being closer to providing a solution to neural rehabilitation of SCI victims [66] to other approaches aiming for a definite cure. 

Unobtrusiveness of robotic devices aiming at upper limb rehabilitation and assisting ADLs is also a key goal within reach of technological advances in this field, which the category of “soft robotics” is aiming at [67]. Body–machine interfaces using neurophysiological signals such as EEG and EMG as input and FES as output to control muscle activity are also under consideration in the context of assistive technologies both for lower limb [66] and for upper limb rehabilitation [68] after SCI. This approach extends the concept of “soft robotics” and “exosuits” to reciprocally incorporate a patient’s body in the control scheme and the assistive technology into the body schema.

### 2.4. Amputation

Nearly 2.1 million people were living with amputation in the United States in 2019, which is 0.63% of the population, and that number is estimated to double by 2050 [69]. Worldwide estimations are not easy to collect largely due to the variability of what causes the amputation, as well as underreporting in less-developed countries [70]. Prosthetics and bionics are usually employed to assist people with amputation [7]. To secure an optimum prosthetic benefit for the traumatic amputees, we need to ensure prompt prosthetic fitting and proper rehabilitation in addition to post-traumatic counselling [71].

Important progress has been made over the past several years in creating prosthetic technologies aiming to restore human functions to those that suffer from the partial or full loss of their limbs [70]. Some of the advances that allow more amputees to have independent lifestyles are in sources of power and electronic controls, socket fabrication and fitting techniques, components, and suspension systems [72]. The rehabilitation for upper limb amputees has also benefited from technological advances such as myoelectric and proportionally controlled devices and elbow joints. For the lower limb amputees, some of the advances that are now used are ankle rotators, electronic control hydraulic knees, energy-storing feet, and shock absorbers [72]. 

Prosthetic types should be selected based on a patient’s individual needs and preferences. There are indications that body-powered prostheses have benefits in durability, frequency of adjustment, maintenance, training time, and feedback, but control can still be further improved. Myoelectric prostheses are more well-established for light-intensity work, and they can improve phantom-limb pain and cosmesis [73]. 

However, many challenges remain, such as the complexity of neural changes after amputation and the still enigmatic fundamentals of motor control in amputees, to understand and address the barriers in patient adoption of technology [70]. Understanding how these critical factors work with device designs and emerging technology can have a great impact on the functional outcomes of patients with limb amputation [72].

### 2.5. Duchenne Muscular Dystrophy (DMD)

DMD is an X chromosome-linked progressive neuromuscular disease that results in muscle fiber degeneration, motor deficiencies [74], and shortened life expectancy [75]. DMD is the most common form of muscular dystrophy [76], with an incidence of 1 out of 4000 male births [77]. The population of people with DMD is expected to grow, due to technological advances that significantly increased their life expectancy [78]. However, their independence-related quality of life remains poor [77].

Robotic exoskeletons have the potential to increase their quality of life by supporting the upper limb to improve their independence [79]. Such exoskeletons can prevent the disuse of the limb and tissue degeneration [80]. Additionally, individuals with DMD need robotic devices for daily assistance and for a significant amount of time [81]. In recent years robotic devices to support the arm [82], the trunk [83,84], and the hand [85,86] of people with DMD were developed and tested with promising results. Novel sensors were used [84,85] in integration with novel robotic designs [83,86] to achieve robust interfacing between the user and the robotic device.

Current studies for the use of robotic exoskeletons in DMD are not many and are limited by a small number of participants [82,87]. More extensive longitudinal studies can give further insights into how DMD affects motor control in different individuals with high functional heterogeneity [88]. Furthermore, novel control interfaces customized to the needs of individuals with DMD need to be developed to achieve robust and intuitive interfacing between them and the assistive devices [74,87]. A complete and multidisciplinary rehabilitation approach may create a favorable environment for robotic rehabilitation in DMD [89,90].

### 2.6. Mental Disorders

Immersive affective applications based on robotics and related technologies have been well under consideration in the study and treatment of psychiatric disorders [42,91]. Immersiveness of affective applications has been demonstrated as a parameter influencing motivation, as well as efficacy of neurotherapy [92,93]. Computational modelling of affect spans already two decades [94,95], and a variety of humanoid or anthropomorphic robots have already been tested in the treatment of cognitive or developmental disorders, among others [91]. Nonetheless, affective and social robots still demonstrate limitations in scope, engagement, and validation, while they are still not universally applied in mental healthcare [96,97]. An approach to improving the affective impact of robotics can be identified in repurposing neurotechnologies in the form of serious gaming [98]. Such an example of repurposed neurotechnologies for affective study can be seen in a 3D graphic environment BCI implemented in UNITY engine [99]. This example is using real-time functional magnetic resonance imaging-based neurofeedback to study emotion regulation—exploring synergistic effects and combinations, which could help develop affective robotics that play a definite role in the multimodal treatment of psychiatric disorders [100].

## 3. Technological Synergies Driving Neural Rehabilitation

### 3.1. Human–Robot Control Interfaces

**Figure 3:**
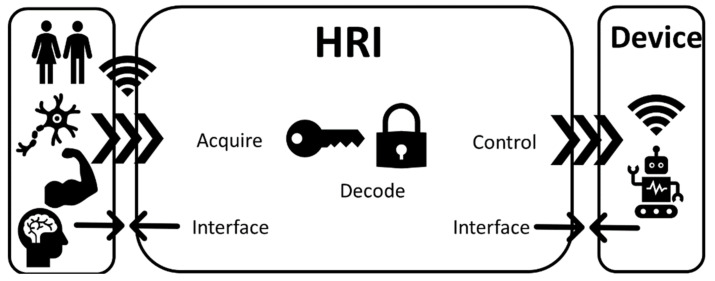
Human–robot interfaces (HRIs) are interfacing the human (brain, muscle, and nerves) with a device by acquiring biological signals, decoding them, and translating them to control commands for various assistive, rehabilitation, or prosthetic devices.

Human–robot control interfaces (invasive and noninvasive) are technologies that acquire, decode, and communicate the intention of a human user to a device via control commands (Figure 3). Such a device can be a wearable exoskeleton or a prosthetic/bionic device [7]. The technologies mentioned in this section interface either directly (digital–neural interfaces) or indirectly (EMG interfaces) with the PNS or with the CNS (BCIs) [101,102]. Such technologies use a physical interface via electrodes that based on invasiveness can be discriminated into either implantable (applied directly at the brain/muscle/nerve) or surface (applied on the skin) devices [102]. 

#### 3.1.1. Digital–Neural Interfaces

Digital–neural interfaces are connections that enable two-way information exchange between the nervous system and the user (Figure 3). Such interactions can occur at various levels, including interfacing among the peripheral nerves and the spinal cord, as well as the brain. In many instances, some of the fundamental biophysical and biological implementation challenges are shared across these levels. Furthermore, such interfaces can be either invasive—such as implantable microelectrode arrays [103]—or noninvasive, such as in the cases of EEG [104] and wireless interfaces. Interfacing with the neural system in an invasive manner for rehabilitation purposes can also be achieved with deeper brain sensing and stimulation. Robotic physical assistance has been converging with deep brain stimulation (DBS), leading to novel forms of neural engineering technology, although this field does not necessarily precisely fit the scope of neural rehabilitation [105]. 

Neural sensing and control interfaces used for the first time back in the 1960s were technologically primitive by modern standards and were mostly focused on prosthetics. Muscles were activated as a group, essentially reducing multiple degrees of freedom (DOF) to a single movement (e.g., grasping). In the decades that followed, several key technological advances relevant to neural rehabilitation have taken place. Fueled by rapid development in digital electronics, microcontrollers, signal processing, control and machine learning algorithms, robotics engineering advanced and synergistically converged with prosthetics engineering, leading to ever more sophisticated artificial limbs. Implantable wireless electronic devices subsequently appeared capable of transmitting signals directly to and from the PNS, avoiding the necessity to maintain an open skin wound. The prospect consequently appeared for mixed-signal assistive electronics to help bypass irreparable trauma to the nervous system by acting as signal amplifier, repeater, and filter.

Interfacing with the PNS via neural electrodes is considered one of the most promising ways to control sophisticated neuroprosthetics [102]. For direct physical interfacing with the nerves, there are two main types of electrodes—namely, extraneural (placed around the nerve) and intraneural (inserted inside the nerve) [101,102]. Extraneural electrodes include epineurial, helicoidal, book, and interfascicular electrodes (which are mainly used for stimulating peripheral nerves), and cuff electrodes, which are capable of both nerve stimulation and recording of neural signals [101,102]. Intraneural electrodes include intrafascicular, penetrative microelectrodes, and regenerative electrodes, which are used for both stimulation and recording [101,102].

At the point that this paper went to press, there is a technological trend toward wireless and less invasive implantable electrodes, as well as toward further shrinking the electrode contact features from the micron to the submicron scale (e.g., carbon nanotubes). Neural cuff electrodes with embedded electronics—which can handle wireless power, data transfer, and have adequate computing power for fundamental signal processing—is, in our opinion, one of the most promising sets of converging technologies. It can potentially provide selective, low impedance signal recording and stimulation without requiring an open skin wound for wire passage.

Despite the aforementioned technological advances, numerous challenges still remain in the application and widespread adoption of digital–neural interfaces. Frequently, significant compromises have to be made regarding the accuracy, response time, and number of degrees of muscle movement freedom [102]. Issues with the powering of neural implants as well as in the wireless transmission of data from and to the implants limit optimal control of external devices [102]. These control issues are further inhibited by the amount of selectivity and resolution that can be currently achieved by existing hardware [102]. Sensing and stimulation electrodes are often a weak link in the signal flow; thus, research and development in the past two decades have increasingly focused on the technological improvement of electrodes and sensors [106]. Additionally, the surgical procedures required to implant neural electrodes, as well as the physical properties of the materials comprising the electrodes, may result in an injury of neural tissue or rejection of the implants by the human body due to biocompatibility issues [102]. Last but not least, due to the novel nature and recent clinical application of implanted digital–neural interfaces, their long-term effectiveness, stability, and reliability are still unclear due to issues related to the longevity of the electrodes [102]. 

#### 3.1.2. Electromyography

To elicit motion, the human nervous system recruits motor units (MUs), motor neurons, and muscle fibers innervated by that motor neuron [107]. The electrical activity of motor neurons can be detected using EMG [108]. An EMG signal is composed of the superposition of action potentials produced by multiple MUs when movement is elicited [109]. Myoelectric interfaces using electrodes can be considered an established technology as they have been proposed since the 1940s and implemented since the 1960s [110]. EMG is routinely used as a tool to indirectly study nervous system motor control organization and function [109] for both healthy and impaired individuals [74,111], as well as a means to acquire data to control assistive robotic devices such as orthoses and prostheses (Figure 3) [112,113,114]. 

EMG uses electrical current to measure motor unit activity noninvasively on the surface of the overlying skin [109] or invasively using needle electrodes and implanted sensors [115]. For surface measurements, low-density EMG is traditionally used, where two sensors are placed closely above the muscle belly [116]. More recently, multichannel and high-density EMG have been developed, where a grid of sensors is deployed over the muscle(s) of interest. Such methods address common shortcomings of regular low-density EMG, including the need for accurate sensor placement [117], and enable EMG decomposition [118]. By decomposing EMG signals, the motor unit action potentials can be reconstructed in vivo without the need to resort to invasive techniques [119]. Lately, implantable EMG systems (iEMG) have been introduced in prosthetic control research [120,121,122] due to their ability to overcome some surface EMG (sEMG) limitations (mitigate the effects of cross talk by specific insertion to the targeted muscle and changes in limb position [122]). However, so far iMEG shows moderate clinical implementation [123]. This may be due to the invasive nature of those sensors. Their chronic implementation is impeded by the small pick-up area and a limited number of MUs measured [123]. However, iEMG is currently being explored for a broad range of applications in robotic rehabilitation for clinical scenarios such as SCI, stroke, and amputation [119,124,125,126], even though for some applications sEMG is still performing better [122]. The development of those measurement methods in combination with a variety of EMG decoding algorithms [124] led to EMG evolving into one of the most common control interfaces for assistive robotics [7] for cases where the residual muscle structure is intact. Additionally, in cases of people with degenerated muscle tissue, EMG showed promising results for interfacing with assistive devices [85]. Even in cases of the complete absence of residual muscle, EMG in combination with targeted muscle and sensory reinnervation (TMSR) and decomposition enabled interfacing with prosthetics for upper limb amputees [125].

Despite the variety of options EMG offers for wearable robotics control, its implementation is in practice limited by multiple factors. Wearable robotics or prosthetics for daily use need to be able to perform unrestricted dynamic motions [126]. Currently, the number of cables and large amplification circuit boxes, especially for high-density EMG, restrict movement, induce movement artefacts, and may result in user discomfort [126]. However, recent breakthroughs resulted in the development of more portable amplification circuitry, which addresses these limitations successfully [126]. Additionally, EMG signal quality heavily depends on electrode placement, movement and cross-talk artefacts, electromagnetic noise, and changes in skin condition [7,127]. This has consequences for human–robot control interfaces as it often results in unreliable and unpredictable control [128], which is further deteriorated outside of controlled lab conditions and can lead to user rejection of prostheses [129]. The development of safe and long-term implantable electrodes can successfully address these issues and improve signal quality for EMG human–robot interfacing [128,130]. Last but not least, the robustness of machine-learning algorithms for EMG is currently limited by the need for retraining [128], significant setup time [131], and the inability to generalize training that happens in a specific spatiotemporal setting [132]. Recently, a new approach using neuromuscular biomechanical modelling for EMG-based human–robot interfacing showed significant advantages over traditional machine learning approaches [131,133].

#### 3.1.3. Brain–Computer Interfaces

BCIs refer to computer-based systems that interface with the brain to acquire, decode, and translate the brain activity of the user into control commands for various devices [134]. A complete BCI framework (Figure 3) does not include only the sensors (to acquire brain activity), but also includes the software (to decode brain activity [135]) and the hardware (to process brain activity and control a device) [136]. BCIs offer a communication method that can be useful when the CNS is impaired by disease or trauma [134]. Depending on the nature of the CNS impairment, BCIs offer a broad set of applications [137], spanning from enabling communication and environmental control to enhancing neuroplasticity and assisting robotic rehabilitation [104,138,139].

The latter presents an exciting clinical application of BCIs, which investigates their use in neurorehabilitation or ADL assistance of people suffering CNS-impairing trauma or disease, or limb amputation [7,104,138,140]. BCIs are often used in combination with exoskeletons to assist robotic rehabilitation [141] or as a means of providing feedback and monitoring recovery [142,143]. Additionally, BCIs can act as neuromodulation techniques exploiting the dormant neuroplasticity of the CNS to promote functional recovery [144] during traditional rehabilitation. BCIs may also be used to enhance neuroplasticity in multimodal combinations with functional electrical stimulation (FES) [145], brain stimulation [146], virtual reality [147], and assistive robotics for motor relearning [145,146]. BCIs show a universal potential as they can address a range of impairments that include stroke [148], SCI [138], and muscular dystrophies [149], while also being able to adapt to their internal variability (i.e., stroke severity). This may increase rehabilitation efficiency (custom-made training regimens and multiple training modes [148]) and reduce costs of rehabilitation (home rehabilitation, lower the burden for rehabilitation physicians). Therefore, BCIs can be a realistic option for ADL assistance or rehabilitation once the associated risks and costs are overcome by their benefits [146].

However, it is still unclear if the therapeutic effects of BCIs are because of their standalone application or due to combination with other therapies [148]. Additionally, there is no significant improvement shown compared to traditional therapy [148], and the generalizability and retainment of BCI rehabilitation effects are still dubious [138]. This may be proven with more studies that include affected subjects as opposed to healthy, with an equal focus to both upper and lower limbs [148], and multidisciplinary protocol designs to assess cost-effectiveness and impact on the quality of life [134]. Improvements in sensor technologies [138] (see Section 3.1) are attempting to solve many of the implementation problems of BCIs, such as complex user interfaces [138] and time-consuming application of gel that hinders the clinical application of BCIs [138], and also to reduce electrical artefacts due to adjacent devices [138]. BCI intention-decoding algorithms (software) are limited by their very lengthy calibration processes and the questionable generalizability between different conditions and people [135,150]. However, the recent rapid development of deep learning, with impressive results [151], is expected to further increase the efficiency of deep learning algorithms for BCIs [135] and their robotic rehabilitation applications [152].

### 3.2. Neuro-Robotics

#### 3.2.1. Exoskeletons

Exoskeletons are devices that aim to interface with the human (Figure 3) and assist with the recovery of the walking function compromised due to sensory and cognitive deficits. Repetitive training using such technological aids assists the human nervous system to create alternative neuron paths to replace the damaged ones [153]. 

##### Technological Challenges of Exoskeletons

Stroke patients who receive electromechanical gait training assistance in addition to physiotherapy are more likely to achieve independent walking [154]. Some of the roadblocks remaining for developing commercially successful lower limb (LL) exoskeletons are human–machine interface compliance, the optimization of the control algorithms, and the smooth coordination with the physiology of the human body with low metabolic energy expenditure [155]. 

The actuation system of exoskeletons is a determinant factor since it affects their design and defines their portability, performance, and effectiveness [156]. There are four main types of actuators used in modern exoskeletons: electric motor (the predominant type), pneumatic, hydraulic, and elastic actuators. LL exoskeletons can be further categorized into assistive and rehabilitation. Assistive are those that assist users to complete ADL that they are no longer able to do due to various impairments. Exoskeletons of this type are frequently controlled with predefined trajectories activated by the patient’s moving intention, and they require high precision control. Most assistive exoskeletons are overground and driven by DC motors, such as Indego [157], eLEGS, AUSTIN, ReWalk, and HAL [156]. The MindWalker exoskeleton [158] has DC motor actuators with series elastic actuators. 

Early upper limb (UL) rehabilitation robotic devices were end-effector type, which means that they were simpler in design and had only one point of attachment to the user’s limb [153]. The InMotionArm of MIT-Manus [159] is attached to the patient’s forearm and is used with robotic therapy games to encourage and synchronize therapeutic tasks, a method widely used in UL rehabilitation systems. The RATULS trial showed analogous clinical effectiveness of robot-assisted training using the MIT-Manus compared to repetitive UL functional task therapy and usual care [159]. Even though these types of end-effector devices for neurorehabilitation are less costly, they are often restricted to mimic the joints, measure joint torques, and drive joint-specific rehabilitation. These limitations are addressed by the use of wearable robotic exoskeletons in an anthropometric fashion. Such devices can be worn by the patient and are attached to multiple locations, permitting a much larger range of movements and the ability to focus on specific joint movements [160]. 

Wearable exoskeletons can be active, with one or more actuators that actively augment the user, or passive, in which case they do not use an external power source and have the ability to store energy and use it when required. Some examples of passive exoskeletons are the Armeo Spring, which allows variable upper limb gravity assistance [161], the GENTLE/s, which is an elbow orthosis setup suspended from the ceiling using cables, and the L-Exos [162], which has a passive forearm DOF. Active exoskeletons aim to assist people with impairments that require further assistance than the one provided by passive due to severity or need for active neurorehabilitation (thus the focus of this work). 

##### Examples of Exoskeletons

Representative examples of active UL wearable exoskeletons include: the NeReBot, which is a cable-driven exoskeleton actuated by three motors that maneuver the user’s arm [153]; the Armeo Power, which supports the rehabilitation of multiple DOF of the arm; and the Exorn, which is a portable exoskeleton developed to support all DOFs of the arm, also containing two at the shoulder and four at the glenohumeral joint [153]. The UL part of the full-body Recupera-Reha [163] exoskeletal system is the latest dual-arm robotic setup designed for stroke rehabilitation. Regarding the distal part of the UL, the SymbiHand finger exoskeleton was designed to provide daily support to patients with DMD [85,86]. Finally, the soft arm exosuit designed for elbow rehabilitation in [164] and the SaeboGlove, which is a lightweight solution for assisting finger and thumb extension, highlight a new and promising technological evolutionary trend toward soft exosuits.

LL assistance-as-needed rehabilitation exoskeletons aim to help users regain functional abilities through repetitive exercise with progressively reduced assistance. Thus, the control is partially predefined and uses online modifications so that it only assists when it is necessary based on patient feedback. Rehabilitation exoskeletons are traditionally stationary and fixed to a treadmill. The Lokomat [165] is an example of a medical exoskeleton for gait rehabilitation that uses electric motor actuators. One of its main advantages is that it can also support severely affected patients to train manually and relieve the therapists from strenuous physical work. Other similar stationary exoskeletons are ALEX with DC motors and PAM with pneumatic actuators [156]. However, the distinctive lines between overground/mobile systems being intended for assistive purposes and treadmill systems being intended for rehabilitation have recently become blurred, as there are also mobile rehabilitation exoskeletons, such as Wearable Walking Helper; Honda:SMA with DC motors; MIRAD, H2, and HAL with electric motor actuators; and LOPES and XoR with pneumatic actuators [156]. In fact, overground systems can provide strong proprioceptive feedback to induce neural plasticity and have been used in combination with treadmill systems in novel rehabilitation protocols [58].

#### 3.2.2. Neuroprosthetics 

Prosthesis (a word that comes from Greek and means addition and attachment) is a device or system that replaces a missing body part to supplement its functionality [166]. Thus, a neuroprosthetic is a device (Figure 3) that interacts with the nervous system and supplements or restores functions in the user’s body [167]. 

##### Technological Challenges of Neuroprosthetics

The purpose of controlled neuroprosthetics is to transfer control intent from the central nervous system to drive the prosthetic devices of users with immobilized body parts [168]. Such control generally requires high levels of concentration by the patient and long training periods, often resulting in high rejection rates of prosthetics [129]. To accomplish robust control, there are two main challenges: (1) development of neural interfaces that last a long period, and (2) skillful control of the prosthetic device comparable to natural movements [168]. Up-to-date hand prostheses are actuated by advanced motors, allowing the restoration of fine motor skills with direct muscular signals connection [169]. In the long run, the goal is to accomplish a quantum-leap advance in neural controllable degrees of autonomy that should permit the user to perform tasks of daily living without effort [168]. Some points to consider regarding control and feedback are the location of the interface with the CNS and the PNS, as well as the invasiveness of the interface, from noninvasive interfaces to the most invasive interfaces requiring surgical implantation [170]. Surgical procedures, such as targeted muscle and sensory reinnervation (TMSR) [171,172,173] and osseointegration [174], greatly improved PNS signal decoding and subsequently improved prosthetic control, as well as the donning/doffing and stability of the prosthetic fixture. In TMSR, motor and sensory nerves of a severed limb are surgically rerouted to reinnervate regions of large intact muscles (the pectoralis for the upper extremity and the hamstring for the lower extremity [171,172,173]). Osseointegration is a surgical procedure where a load-bearing implant is directly integrated with the residual bone of the amputee to improve prosthetic connection and sensory feedback [174]. 

Regarding control, the key technologies used for interfacing prosthetics with an amputee are EMG, neural and BCI interfaces (interfacing directly or indirectly with the PNS), and body-powered and impedance/admittance control (interfacing with the residual/unimpaired anatomy of the user) [7,102,168]. The three key ways to interface with the PNS/CNS are described in Section 3.1. and are very popular with UL prosthetic control [7]. Impedance control manages the relation between position and force and is mainly applicable in LL prosthetics controlled by the multi-joint mechanical impedance. To control a prosthetic, it is equally important that the user can have a proper feedforward and feedback signal. This can be achieved via body-powered control methods that focus on the use of the remaining anatomy of the human to mechanically control a prosthetic limb [7]. Body-powered control can be complemented/combined with other approaches such as cineplasty, muscle bulging, myoelectricity, and myo-acoustics to create hybrid control interfaces for UL prosthetics. Additionally, FES based neuroprostheses (in the case of FES, the impaired existing limb is considered the prosthesis) can stimulate muscles or nerves [175,176] to elicit movement in the impaired limb and enable UL function restoration [177] and LL gait training [178]. FES can act as a standalone [177,178] or in combination with robotic exoskeletons [179]. 

Some of the most common decoding algorithms for neuroprosthetics control can be categorized as (1) independent models, (2) dynamic models, (3) reinforcement, and (4) classifiers. In the first category we have algorithms such as, Bang-Bang control, which is activated when a specific limit for a measured variable is reached, used in cases such as the delivery of cortical electrical stimulation [180] and the mapping of stimulus thresholds in high electrode count implanted neurostimulators [181]. A finite state machine contains the measurement of a system variable, which in combination with the modelled system’s present state activates an action and a state shift [182] and is used in periodic functions such as the gait during walking. A population vector algorithm is based on the fact that there are directional preferences in different neurons [183], and it can be applied for the cortical representation of the arm motor control, encoding the lengthening or shortening of specific muscles. In the second category, there is the Kalman filter—which is a recursive optimum estimator and is mostly used for taking out signal from noisy measurements [184], variants of which have been proposed for neuroprosthetic closed-loop control to capture features of the neuroprosthetic task—and the point process filters—where the activity of individual neurons can be modelled as point procedures [184]. The reinforcement learning scheme may also be suitable to neuroprosthetic control in a real-life usage scenario where the task and related trajectory varies and accomplishing the task may be the only reinforcement signal offered [185]. Finally, in the last category are artificial neural networks, which are a data-driven method arranged in layers with neurons or nodes that can be used to achieve control of a myoelectric prosthetic hand [186], and support vector machines, which are supervised machine learning methods that can implement regression or classification.

##### Examples of Neuroprosthetics

For UL amputees, existing commercial prosthetic hands offer single-DOF actuator designs to open and close the fingers and thumb, such as Ottobock’s Sensorhand Speed, products from Motion Control Inc. and RLSSteeper Inc., or multiple-DOF actuator designs with articulated fingers, such as the Touch Bionics i-LIMB and the BeBionic hand [9]. The Otto Bock Michelangelo hand is a combination of fully articulated and single-DOF hand-design [9]. More actuated DOFs, various grasps, and control mechanisms are provided by several intrinsically actuated (actuation, transmission, and control elements are embedded in the prosthetic) prosthetic hands. Such hands are the Fluidhands, the DLR hands, the Cyberhand, and the Smarthand [9]. The need to transmit sensory feedback from the prosthesis [187] led to the development of the Modular Prosthetic Limb (MPL) with 26 articulated and 17 controllable DOFs with bidirectional capability and the DEKA arm, which provides powered movement complemented by surgical procedures (such as TMSR) for sophisticated control over multiple joints [8]. Other successful examples of prostheses that allow sensory feedback to enhance motor control include Revolutionizing Prosthetics, the HAPTIX, the Cyberhand, and the NEBIAS [169]. Lastly, applications of additive technologies in the manufacturing of prosthetic limbs, such as rapid-prototyping (3D printing), are becoming an integral part of UL prostheses [188], with commercial outcomes such as the Robohand and the Andrianesis’ Hand [189]. 

For LL amputees, neuroprosthetics assist with movement and balance [8]. While passive devices offer only basic functionality, semi-active prostheses are capable of adapting their behavior. They achieve this by controlling magnetorheological systems (Rheo knee, Ossur) or valves (C-Leg, Ottobock) with information from the gait cycle [190]. Active or powered prostheses are actuated by motors and provide greater performance and functionality. Various research groups are developing powered knee, ankle, or leg prostheses that provide kinematics that are similar to able-bodied movement in a more effective way than passive and semi-active systems [191]. LL prosthetics that control both knee and ankle joints are the Vanderbilt Prosthetic Leg by Center for Intelligent Mechatronics, the OSL by Neurobionics Lab, and the AMPRO by Advanced Mechanical Bipedal Experimental Robotics Lab [191]. Prosthetics of the knee joint with impedance control have been developed by Biomechatronics Group, Massachusetts Institute of Technology, and Delft University of Technology [190]. Other commercial examples of bionic ankles are: the MIT Powered Ankle, the first commercialized powered ankle prosthesis by Ottobock and previously by BionX; the one from Arizona State University, commercialized by SpringActive; one from the Mechanics Research Group (Vrije Universiteit Brussel), commercialized by Axiles Bionics; and one from the Biomechatronics Lab (Stanford University), commercialized by Humotech [191]. Several 3D printed LL prosthetic designs are being fabricated by companies, such as the bionic leg prostheses by BionX Medical Technologies, the Exo-Prosthetic leg, and the Andiamo leg [192].

### 3.3. Virtual and Augmented Reality

VR is an artificial simulation or a reproduction of a real-life environment using immersive projections of virtual objects [193]. Furthermore, AR is a technology that projects layers of computer-generated graphics in real world space, while mixed reality (MR) allows for physical interactions of virtual and real elements [194]. These virtual reality environment (VRE) technologies have opened new possibilities for effective neural rehabilitation through accurate feedback and presentation [195]. However, while they have existed for approximately two decades in the form of applied research, they have yet to be fully integrated into mainstay neural rehabilitation [196,197,198]. A primary objective of neural recovery is for patients with motor disabilities to reacquire the ability to perform functional activities. Repetition, encouragement, inspiration, and task-driven preparation can promote successful recovery [199]. 

Contemporary literature on motor control indicates that enhancing functional tasks may benefit from task-oriented biofeedback therapy [144,200,201]. Task-oriented biofeedback can be described as the use of instruments to engage subtle physiological processes while integrating task-dependent physical therapy and cognitive stimuli [202]. Furthermore, VR/AR environments can significantly help task-oriented biofeedback and robotic-assisted rehabilitation by providing visual, auditory, and physical interaction in an immersive manner. The key underlying hypothesis in this direction states that virtual-trained skills and functional gain can also transfer to the real-world, which has been demonstrated for advanced motor skill acquisition in healthy motivated individuals [203]. A critical point toward applying this concept for rehabilitation of motor-impaired individuals lies with ecological validity. Until recently, VRE technology applications in neural rehabilitation have been limited to proof-of-concept, novelty, and immersion improvement techniques, yet the question of whether they provide added value in generalized real-world settings still remains unanswered [197]. 

Advances in technology and affordability, as well as their advent popularization, allow for critical study of the aforementioned systems’ efficacy and ecological validity [198,204]. As such, the novelty and immersion factors related to VRE have been proven to promote motivation, excitement, and task engagement, subsequently leading to increased efficacy of virtual rehabilitation regimens over traditional forms of rehabilitation [198]. Furthermore, the physiological mechanisms of presence and immersion (which we discuss below) provide added value to motor function gain well beyond the benefits of increased motivation [204]. 

It should be noted that multimodality and synergistic effects of VR with other novel neural rehabilitation technologies have also been recently shown in patients with motor impairment due to SCI [58] and that the neural plasticity effects, while demonstrated, are still under investigation [65]. Task-oriented biofeedback using VR/AR systems in conjunction with robotic-assisted rehabilitation can improve patients’ recovery. For example, L-EXOS is an exoskeleton that covers the full spectrum of human arm movement by integrating a wearable structure with an anthropomorphic workspace that offers five DOF. L-EXOS uses a VR technology for task-oriented therapy in the form of a task-oriented exercise program mission [162]. Additionally, Tageldeen et al. designed a VR-based serious game for arm rehabilitation using a wearable robotic exoskeleton. Their VR serious game goal is to improve the patient’s motivation for repetition, which is critical in a patient’s therapy [205]. Comani et al. integrated high-resolution EEG (HR-EEG) recordings, a passive robotic device, and VR for stroke recovery. The robotic device works with five VR task-driven training applications and is synchronized with an HR-EEG system. This set-up enabled them to acquire EEG signals in association with the execution of specific training tasks to quantify the task-related changes in the brain activation patterns during recovery of motor function [206]. 

Furthermore, VR/AR systems without robotic devices attached have also been demonstrated to offer significant gains in neural rehabilitation [202]. For example, in a VR system designed by Kynan Eng et al., the patient is seated at a table facing a monitor that projects virtual arms in the same orientation as their own. The goal of this exercise is to maximize a point score by hitting, catching, and grasping virtual balls. During therapy sessions, the patient’s real arm’s movement is correlated to the virtual arm’s movement to encourage the patient to treat the virtual arm as part of their own body [207]. Sucar et al. also suggested another gesture therapy using a VR system, in which they developed a VR-based motor rehabilitation therapy that enhances gestures. Within a secure virtual world, the patient is challenged to perform everyday tasks in the form of brief, intense games [199]. On the other hand, AR-based systems can provide help during task-oriented rehabilitation therapy. For instance, YouMove is an AR system using an AR mirror that reflects the patient’s movement and provides feedback and guidance. The system trains the patient through a series of stages that gradually reduce the system’s guidance and feedback [208]. Another noteworthy AR-based motor therapy protocol was designed by Hondori et al., in which they designed a spatial AR system for hand and arm movement [209]. The device monitors the hand of a subject and creates an audio–visual interface for rehabilitation-related activities requiring gestures of the wrist, elbow, and shoulder. It tests the length, speed, and smoothness of movement locally and can send the images and data in real time to the clinic for further evaluation. 

In conclusion, VR/AR systems for neurorehabilitation offer significant results to the therapy process due to the feedback they provide to the patient. Additionally, task-oriented training and gamification of therapy provide the motivation necessary for the patient to perform repetitive tasks. This convergence between gamification and task-oriented training is one of the core components in VR/AR systems, thus making them a promising modality for neurorehabilitation and home-based therapy systems. There are, however, certain challenges in VR/AR-based rehabilitation therapies that need to be overcome. There is theoretical ambiguity and immaturity in clinical VR research for the contexts of immersion and presence, where the terms are mistakenly used interchangeably [210]. Immersion can be achieved by delivering inclusive, extensive, surrounding, and vivid illusion of reality to the end user [211]. Presence, on the other hand, refers to the sense of being within a simulated environment [212,213]. The sense of presence is boosted by the vividness of the simulation—which leads to immersion—and by the interactivity between the user and the environment [213]. In addition, reducing motion sickness and discomfort ought to result in improved interactivity [214]. This can be achieved by optimizing image processing, which will boost framerates and minimize system latency [215].

### 3.4. AI Algorithms 

#### 3.4.1. AI Algorithms for Human–Robot Interaction

Human–robot interaction (HRI) is a diverse research and development field that involves artificial intelligence, robotics, and the social sciences for the purpose of interaction and communication between humans and robots. It has a wide range of applications, such as industrial robots, medical robots, social robots, automated driving, search and rescue, and several more [216,217]. In this paper, the focus of HRI is on medical robots and, more specifically, in those employed for neural rehabilitation applications. 

Neurological disorders are globally one of the leading causes of disability and death, representing a severe public health problem [218,219]. Robot-aided neural therapy systems assist in addressing this significant issue by helping patients heal more quickly and safely. Interaction between a patient and a robot is not a trivial task, and a wide range of issues need be addressed, such as safety, learning by demonstration, imitation learning, cognition and reasoning, perception, and sensation, etc. [220]. Most of these issues are typically handled by AI algorithms and systems that improve the overall interaction and experience of the patients. Due to multiple representations in an environment, there is usually an excess of multimodal data, such as visual, audio, infrared, and ultrasound, which are used as input in AI algorithms capable of performing object classification, prediction, and task planning tasks [221]. Bayesian models, hidden Markov models, and genetic algorithms are some examples of widely used AI algorithms that routinely perform such tasks. Recently, the application of machine learning and, more specifically, deep learning (DL) have demonstrated promise for significant performance increase; thus, this paper will focus on DL.

An essential task for most robotic systems is object classification (OC), during which the robot classifies features and attempts to perceive its environment. Currently, convolutional neural networks (CNNs) are considered part of the state-of-the-art among AI models for OC [222]. Because of their topology architecture, CNNs can capture both low- and high-level features in visual data. A promising CNN model is PointNet, which is based on point clouds, an important type of geometric data structure. It provides a unified architecture for applications ranging from object classification and part segmentation to scene semantic parsing, all of which are applicable in robotic systems [223]. One of the most widely used and state-of-the-art CNN models for OC is the “You Only Look Once” (YOLO) model, which uses a different approach from most CNN models [224]. The entire image is used as input to the CNN, which separates it into distinct regions and estimates the bounding boxes for each one. The YOLO model requires just a single forward propagation through the neural network to generate predictions, thus it “only looks once” at the input image. The algorithm makes sure that the OC algorithm only detects each object once and then outputs recognized objects along with their bounding boxes. 

Another important task for robotic systems that interact with humans is natural language understanding (NLU), in which a human operator commands the robot through natural language. One of the state-of-the-art artificial neural network (ANN) architectures for NLU are recurrent neural networks (RNNs), which are based on the idea that decisions are influenced by past experiences. In other words, RNNs allow previous outputs to be used as inputs while considering hidden states. A state-of-the-art model for robotic NLU is the Mbot [225]. In particular, Mbot uses an RNN with long short-term memory (LSTM) cells to perform action detection and an RNN with LSTM cells to perform slot filling. The action detection network identifies corresponding activity associated with the natural language commands, while the slot filling network assigns labels to all words and identifies slots such as object, destination, source, sentence, and person.

Another key component in almost all robotic systems is action planning. In machine learning (ML) the most promising approach for action planning is reinforcement learning (RL). RL refers to goal-oriented algorithms, which learn how to attain a complex objective (goal) or how to maximize a particular function over multiple steps; for instance, RL networks can maximize the points won in a game over several moves. These algorithms are penalized, like a pet incentivized through scolding and punishment, when they make the wrong choices and are rewarded when they make the correct ones (hence the concept of reinforcement). One of the modern ANNs that leverage RL are deep Q-Networks (DQNs), which are based on the idea of Q-learning [226]. In Q-learning, a robot learns a policy of how to take an action at a particular state. Google Deepmind has developed a DQN that discovers end-to-end policies directly from raw pixels: it is capable of solving more than 20 tasks in various simulated physical environments, such as pendulum swing-up, cartpole swing-up, balancing, and object grabbing, etc. [227].

To conclude, robot perception, command processing, and action planning are key components in rehabilitation robotics and HRI, which are enhanced by DL models. However, DL models require substantial computational resources and powerful hardware to run efficiently, which makes them less preferable in mobile robotics and wearable applications. Nonetheless, quantum ML is an emerging and rather promising field that could potentially provide solutions to the computational costs challenge of classical ML [228]. As has been demonstrated, quantum ML can dramatically decrease computation complexity in certain algorithms, such as principal component analysis (PCA): in classical ML we face complexity with a big-O notation factor of *O(d^2^)*, while in quantum ML we face *O((log_d_)^2^)* [229]. Thus, quantum ML can provide DL/ML algorithms with an advantage to leverage even more data and tackle even more complex computations. 

Another concern regarding AI algorithms is safety risks due to the uncertainty of the robot’s actions under particular circumstances. So far, very few exoskeletons have secured Food and Drug Administration (FDA) approval for in-home use, and it is rather important to design rehabilitation robotic devices with tight and demonstrable safety standards to raise their trustworthiness in the consumer base. One emerging field that could potentially provide solutions to safety concerns, due to the nature of the materials used for the fabrication of mechanical components, is soft robotics [230]. The field has been highlighted as an emerging research field by the US National Science Foundation [231].

#### 3.4.2. AI Algorithms for Neural Signal Processing

With the growing complexity and scale of neural recordings, the role of neural signal processing has been gaining significance in the field of neurorehabilitation. The aim of neural signal processing is to process and analyze neural signals from which useful information and insights about the brain’s information processing will be extracted and transmitted through neuronal ensembles. Reading out neural signals is significant to transmit neurofeedback to the brain or computer devices that support communication through brain–machine interfaces (BMIs) in neural engineering. Furthermore, assessing neural feedback, monitoring/decoding brain activity, and controlling prosthetics are key components for BMIs used in neural rehabilitation. Therefore, BMIs that are involved in robot-aided neurorehabilitation can benefit the most from increasingly accurate neural signal processing because of the significance of neurofeedback involved in neural therapies [232]. 

Neural signal processing involves an algorithmic framework that is often divided into the following stages: signal capture, signal preprocessing, and main processing [232]. In signal capture, two approaches (invasive and noninvasive) are used to acquire information. The invasive approach includes significant modalities such as electrocorticography (ECoG) [230], microelectrodes, and multiple electrode arrays [233,234]. In the case of noninvasive techniques, approaches are loosely divided into two groups: contact and remote methods. EEG is a contact-based technique, magnetoencephalography (MEG) is one of the remote-based methods, along with functional magnetic resonance imaging (fMRI) [235]. During the preprocessing stage, the signal is converted into a digital format, denoised, and imported into a signal processor. Furthermore, during the main processing stage, an essential algorithmic step is spike detection. A popular approach for spike detection is using wavelet transform (WT) but also advances in ML bring promising results using CNNs [236,237]. After spike detection, feature extraction is done mainly using PCA [238] or local Fisher discriminant analysis (LFDA) [239]. The final step during neural signal processing is feature clustering. There are many unsupervised clustering algorithms that can be used in feature clustering, and one of the most common is the *k*-nearest neighbor (*k*-NN) [238]. A clustering algorithm commonly used in neural signal processing is the expectation-maximization (EM) algorithm [240]. 

The application of the aforementioned AI-based algorithmic framework aims to boost the accuracy of signal processors, which form an integral part of neural biofeedback-based rehabilitation and assistive device control tasks. For instance, a CNN developed for feature extraction and spike detection in epileptic cases using EEG recordings outperformed all other tested models, achieving 0.947 AUC (compared to, for instance, support vector machines with a Gaussian kernel, which achieved an AUC of 0.912 [237]). In addition, a combination of LFDA and a Gaussian mixture model was implemented by Kilicarslan et al. to decode a user’s intention and control a lower-body exoskeleton. Their decoding method, when tested on a paraplegic patient, presented ~98% offline accuracy and short on-site training (~38 s) [239]. Furthermore, HEXOES is a soft hand exoskeleton that helps post-stroke patients in ADL by using EMG signals from the left hand to coordinate movement of the right hand. An artificial neural network was trained to decode the neural signals and extract the combinations from the left hand in real-time, achieving a low 8.3 ± 3.4% validation error [241].

In final analysis, neural signal decoding in real-time is a rather tough engineering challenge for all BMIs involved in neural rehabilitation and needs to be rather accurate to boost the BMIs’ effectiveness and usability. However, most of these algorithms can be successfully applied only on a limited number of neural signal inputs, in some cases only on one type. Therefore, an algorithmic framework for processing all types of neural signals in parallel is needed. Neural parallel processing is an emerging and rather promising field that can significantly boost neural signal processing tasks. In neural parallel processing, advanced algorithms are used for spike detection, feature extraction, and clustering in all parallel signals, such as exponential-component–power-component (EC-PC) spike detection and binary pursuit spike sorting [242]. Because of the large amount of data and heavy computation required, graphic processing units (GPUs) are used for massively parallel data processing [243].

## 4. Conclusions and Future Directions

**Figure 4:**
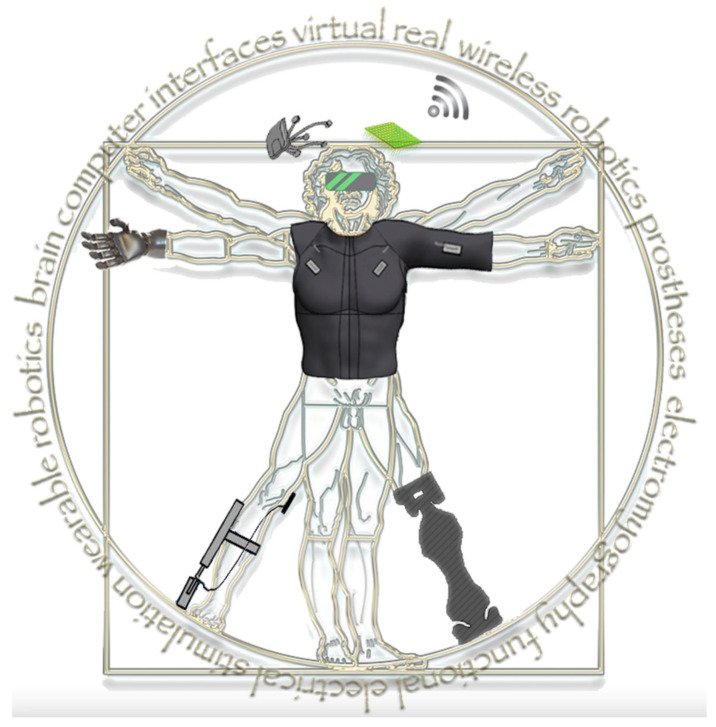
Convergence of key technologies will synergistically enable complex applications of neural rehabilitation and improve outcomes of patients with disabilities.

In this article we have reviewed key robotic technologies that facilitate neural rehabilitation and discussed roadblocks in their development and application, as well as presented current and emerging directions in the field (see Table 1). These technologies feature different points of origin but address clinical challenges and generate synergistic added value in the field of neural rehabilitation. This continuous evolution and convergence, as well as occasional inventions that have a particularly strong impact in the field, are likely to lead to an amalgam of neural rehabilitation assistive technologies whose utility increasingly exceeds the sum of its parts in terms of applications and outcomes (Figure 4).

During this ongoing transformation of the field, certain technologies stand out with respect to the relative advantages they offer. Through a natural paradigm shift, neural interfaces can now be considered as connections that allow information to be reciprocally exchanged between robotics and the human nervous system. As such, BCIs can provide decades-worth of experience in signal recording from the central nervous system, interpretation of motor and sensory outputs, as well as modulation of its functionality. Retraining CNS circuits and promoting adaptive neural plasticity have been recognized among key principles of neural pathways repair by the US National Institute of Neurological Disorders and Stroke [244]. 

Direct digital–neural interfaces and peripheral neurophysiological recordings, such as EMG, can provide pinpoint accuracy, increased resolution, and quality of neuronal signal detection, as well as the ability to exert command control. Exoskeletons, prostheses, wearable and soft robotics, rapid prototyping technologies, and electrical stimulation each demonstrate the unique ability to overcome neurological impairments and bridge lesions according to their applications and indications. These man–machine interfaces and robotics provide a way to deliver effective rehabilitation to, i.e., stroke survivors as a standalone modality or in combination with traditional rehabilitation [7,14,15,24]. This is mainly due to their ability to perform repetitive rehabilitation, adjust the intensity of rehabilitation, use a standardized training environment, and reduce the physical burden of physiotherapists [1,2].

As scientific analysis and understanding of human motor control advances in tandem with modern electronics and computational power, the pursuit of more disruptive therapeutic interventions has become possible. For example, the use of robot-driven locomotor perturbations in order to manipulate an individual’s motor control strategy for therapeutic purposes is a radical and particularly promising new such approach [245,246]. Such approaches also offer novel insight into human motor control and adaptation that need to be taken into account during the design of protocols for robot-assisted gait rehabilitation [247]. Advances in wireless transmission and battery technology facilitate their overall networking and autonomy. Finally, our understanding of embodiment has led to the integration of virtual, augmented, and mixed reality techniques to improve the registration of the aforementioned technologies and devices into a perceived body schema. Body and CNS scheme modification and body area networks work together to overcome disability. Examples can be identified with the mirror box and rubber hand illusions, both intriguing cross-modal illusions used in clinical neurorehabilitation that employ multisensory integration and that are also used to improve neuroprosthetics [248]. Furthermore, VREs offer a unique mode for multisensory integration, taking advantage of increased immersion and presence to deliver neural rehabilitation regimens [197].

Furthermore, fundamental lower-level technological improvements such as advances in software and hardware speed, accuracy, computational power, power autonomy, and the capacity to learn from data (machine learning) can be reasonably expected to continue offering incremental contributions to neural rehabilitation assistive technologies at an ever-increasing pace. To achieve further synergies, a multidisciplinary approach is essential: traditional research in physics and engineering must now work alongside chemical, biological, and medical science to develop new applications and acquire new capabilities [249]. As an example that stands out, it is worth considering that multiple immersive man–machine interfaces have been demonstrated to synergistically promote dormant neural plasticity and patient functional recovery even in chronic conditions previously thought irredeemable, such as in cases of chronic complete SCI [58,65]. 

System-on-chip integration has been revolutionizing the integration of analogue, digital, and radio frequency (RF) electronics on a monolithic piece of silicon during the past four decades. This technology has been merging with biologically targeted MEMS and creating new possibilities in the design of biochips, electrodes for neural interfacing, low-power implantable systems, and energy harvesting solutions. Apart from the obvious benefits of crafting less invasive and more effective electrodes, such technological convergence may also facilitate improvements in on-chip power transmission coils, so that power to implantable systems is supplied noninvasively from outside the body, in tandem with a wireless data link. 

These advances in electronics integration are not devoid of obstacles and roadblocks. Massive parallel distributed processing of information was once heralded to be around the corner, yet the lack of reliable, nonvolatile analogue on-chip memory has proven to be a formidable technological roadblock (though recently discovered memristor electronic components may finally offer a way around it). Furthermore, inherently two-dimensional techniques used to fabricate modern electronic chips, as well as noise and heat distribution problems, have placed limits to further miniaturization and power density concentration offered by deep sub-micron system-on-chip fabrication technologies. 

Three-dimensional printing is clearly a socioeconomically disruptive manufacturing technology, with significant impact in several areas of biomedical engineering, including neural rehabilitation. Blueprints can be turned into 3D objects in minutes utilizing a variety of materials: thermoplastics, resins, ceramics [250], adhesives, metals [251], food stuff, biological tissue [252,253], and many others. Apart from the obvious advantages of rapid prototyping and distributed manufacturing capability, 3D printing has already been having a significant impact in several ways. It leads to improved volumetric efficiency [254], increased elasticity, and deformity tolerance of manufactured objects, such as in the case of implantable biomedical devices [255], wiring, sensor, and chip embedding and device-level component integration [256].

This convergence of multi-composite and 3D printing technologies could be exceptionally valuable in neural rehabilitation, particularly in the creation of novel, flexible, personalized implantable sensing and targeted drug delivery devices, as well as for developing soft exoskeletal robotic aids [255]. Bioprinting involves 3D printing of structures using biological substances, including mixtures of cells, stem cells, and growth factor solutions. It is another promising point of technological convergence with respect to flexible 3D printing, wireless data and power transmission, and energy scavenging electronics [256]. The prospect of combining a body’s own cultured cells with biocompatible materials in order to build multi-composite implantable devices could potentially revolutionize data acquisition and stimulation inside the human body, which would be particularly valuable for neural rehabilitation applications.

On the software side, the most significant impact may be expected from the field of artificial intelligence, specifically machine learning. Right now, when designing devices directly interfacing with the peripheral nervous system, there is a tight balance between optimizing the electronic and mechanical properties, while facilitating surgical implantation and post-surgical signal processing adjustment. By opting for multiple nanoscale-level electrodes, possibly distributed around the neural tissue in a loose “smart dust” paradigm [257,258], it may become possible to distribute the electronics–neural points of interface. Such a design choice would make the system less dependent on precise surgical placement and more robust with respect to tissue shifting (specifically the movement of muscles). However, increased uncertainty of placement would also present a new challenge in calibrating and integrating the signal. This is the point where AI-based automation algorithms may be able to significantly contribute to reducing calibration and adjustment time so that the signal can be extracted, amplified, and interpreted efficiently and consistently no matter the placement distribution of the miniaturized distributed electrodes on the nerve.

We may choose to identify certain key future milestones in this continuous technological transformation. Conceiving the electromagnetic fields and waves generated by the human nervous systems as continuous and integral to the rest of the electromagnetic spectrum will help support and brainstorm novel approaches to signal acquisition, data flow, and information processing. Such systems could be communicating with the nervous system on biological terms and with devices on electronics terms utilizing closed feedback loops [259,260]. Nervous signals will adjust a system’s parameters to optimize performance and furthermore, attempt to induce functional and structural changes in the nervous system, while system parameters will be constantly readjusting based on measurement results. Such a loop would require searching the parameter space with AI algorithms for efficiency, a task at which genetic algorithms have been demonstrated to excel [261,262]. On a hardware level, this procedure may ultimately lead to the development of true “neural dust”, a free floating, swarm-level, AI-based, self-adjustable interface with the nervous system for seamless acquisition and control [257,263,264]. Long before that is possible though, we may expect multiple effective local area body networks with implantable and noninvasive devices. These will communicate with various interface nodes of the nervous system, bridging lesions and promoting targeted neuroplasticity [265].

Finally, despite technological progress, innovative applications, synergies, demonstrations of feasibility, and a widening of the clinical base, in order to produce undisputable benefits for the patients, the field needs to successfully tackle clinical translational challenges that are impeding a lot of solutions to make a real impact. While identifying challenges and translational roadblocks is the primary key to focusing research efforts, prospective design of improved protocols, trials, interventions, and outcomes could be the key to achieve and demonstrate clear benefit [266,267,268]. 

## 5. Limitations

The selection of the narrative review format, instead of a more systematic review approach, was motivated by our intention to discuss key technological synergies in a broad, flexible, and comprehensive fashion. The methodology of a systematic review approach would require a much tighter focus, which would be incompatible with the objectives of this paper. A narrative review format allows us to discuss the gap between robotic rehabilitation and various key technologies from which robotic rehabilitation can benefit and speculate on their integration and future directions. This approach can also better accommodate personal insights on synergistic technological convergence, leveraging the diverse mixture of expertise of the authors. On the other hand, lack of systematic methodology does not allow for acquiring the highest current level of evidence for most of the conditions and technologies discussed.

Some other clear limitations of a narrative review include the fact that it does not ensure an unbiased cover of the scientific literature by locating all relevant literature but instead discusses pivotal articles known to the authors. However, we refer to a great number of studies from various disciplines and research groups, and in this way we attempt to avoid fixation of the discussion to a specific research group, institution, or trending theory. Instead, we discuss every technology, including various viewpoints and criticisms.

## Figures and Tables

**Table 1:** Summary of all the technologies discussed in this review and their area of application, readiness level, and major roadblocks. Additionally, we highlight the existing interaction between those and propose beneficial potential interactions.

Robotic Rehabilitation Technology	Underlying Technologies	Area of Application	Readiness Level	Major Roadblocks	Convergence
**Human–Robot** **Control Interfaces** **Section 3.1** **Technologies that enable the communication between humans and neuro-robotics**	**Digital–Neural ** **Interfaces** **Section 3.1.1** Technologies that interface the human nervous system with robotics.	Implantable devices acquiring data, stimulating nerves, and assisting signal transfer across traumatized parts of the central and peripheral nervous system.	Microelectronic design processes are mature. Microelectromechanical systems (MEMS) design processes are well developed; however, simulation design tools need improvement.	Parts of the CNS are not easily accessible to implantable devices without traumatic and risky surgical procedures (invasiveness). Electrode and implantable device placement require precise navigation inside the human body, with error tolerances that are not always possible to achieve. Selectivity of electrodes, data flow, and long-term consequences are still active challenges.	*Existing:* Microelectronic very large-scale integration (VLSI), microelectromechanical systems (MEMS) design and micromanufacturing techniques lead to novel electrodes. Low power mixed signal and radio frequency (RF) electronics design leads to increasingly more power-autonomous implantable devices. *Potential:* MEMS microgenerators lead to complete power autonomy for implantable devices.
	**Electromyography** **Section 3.1.2** Enables the communication between a user and a robotic device by interfacing with the muscles.	Rehabilitation of stroke, muscular dystrophy, and amputation.	There are existing commercial applications. Ongoing research toward musculoskeletal modelling and safe and reliable implantable devices.	Lack of portability restricts movement and limits convergence with robotics. Unreliable sensors’ result in limited interfacing, which further deteriorates outside of lab conditions. Intention decoding algorithms for electromyography (EMG) are of limited robustness and generalizability.	*Existing:* Virtual reality, functional electrical stimulation, robotic exoskeletons, prosthetics. *Potential:* EMG needs the development of reliable sensors and portable amplifiers. Safe and reliable implantable sensors may achieve better signal quality. Additionally, better AI algorithms or biomechanical models for intention decoding may result in improved human–robot interfacing.
	**Brain-Computer Interfaces Section 3.1.3** Enables the communication between a user and a robotic device by interfacing with the brain.	Rehabilitation of stroke, spinal cord injury, muscular dystrophy, amputation, traumatic brain injury, and mental disorders.	There are existing commercial applications. Research is still underway toward better sensors and intention decoding algorithms.	Unclear therapeutic benefits compared to traditional rehabilitation. Sensors still require long set-up times. Intention decoding algorithms require long calibration and lack generalization.	*Existing:* Virtual reality, functional electrical stimulation, robotic exoskeletons, and prosthetics. *Potential:* Brain–computer interfaces (BCIs) can improve dramatically with the use of better digital–neural interfaces and the development of AI algorithms for intention decoding.
**Neuro-Robotics** **Section 3.2** **The science and technology of embodied autonomous neural systems.**	**Exoskeletons** **Section 3.2.1** Assists with the recovery of function compromised due to sensory and cognitive deficits or daily assistance.	Rehabilitation of stroke, spinal cord injury, muscular dystrophy, traumatic brain injury, and mental disorders.	There are existing commercial applications. Research toward soft exoskeletons is currently attracting interest.	Human–machine interface compliance, optimization of the control algorithms, and the smooth coordination with the physiology of the human body. Restrictions to mimic the gesture of the joints, to measure joint torques, and to drive joint-specific rehabilitation.	*Existing:* BCI, virtual and augmented reality, AI algorithms. *Potential:* Sensors for new information on human intent and motor status, big data for the vast number of physiological signals, machine learning for new control approaches, and 3D printing of materials for customization and cost-effectiveness.
	**Neuroprosthetics** **Section 3.2.2** A device or system that replaces a missing body part to supplement its functionality.	Amputations.	There are existing commercial applications. Ongoing research toward better fitting and human–machine interfacing solutions.	Patient’s reaction to long-lasting implantation of microelectrode as well as the proper part of the body to collect a signal. Limitations in stretchable electronics, electrode–skin interfaces, and personalization.	*Existing:* BCI, virtual and augmented reality, AI algorithms. *Potential:* Control methods that include peripheral nerve interfaces and BCIs. Decoding and control using biomechanical musculoskeletal modelling and model-free machine learning.
**Virtual Reality and Augmented Reality** **Section 3.3** **Technologies that generate an artificial simulation of an environment (virtual reality, VR) and project computer generated graphics in real world space (augmented reality, AR)**	**Virtual Reality** The combination of algorithms, sensors, and high-definition (HD) display for the reproduction of an environment using virtual objects. **Augmented Reality** An augmented version of the actual physical world that is accomplished by the use of visual objects, sound, or other sensory stimulation.	Task-oriented biofeedback therapy, rehabilitation of stroke, brain, and spinal cord injury.	There are existing commercial applications. Research toward the “interaction” aspect of VR and AR.	Theoretical ambiguity for presence and immersion. Motion sickness and discomfort is another roadblock.	*Existing*: Artificial intelligence, human–computer interactions (HCIs). *Potential*: Improving VR/AR system’s latency may increase interactivity. Additionally, a solid theoretical basis for presence will give clear direction for the fields.
**AI Algorithms** **Section 3.4** **Algorithms that learn from experience and simulate human-level intelligence.**	**AI algorithms for Human–Robot Interaction** **Section 3.4.1** Deep learning algorithms aimed to enable and enhance the interaction between humans and robotic devices.	Object classification, action detection, and action planning.	There are existing commercial applications. Research on quantum machine learning (ML) and safety measures is still underway.	Computational complexity, computing resources, and safety risks.	*Existing*: HCIs, virtual reality, robotic exoskeletons, and prosthetics. *Potential*: Quantum ML may overcome the computational challenges of the current ML approach.
	**AI algorithms for Neural Signal Processing** **Section 3.4.2** Machine Learning and Unsupervised Learning algorithms and techniques that process and analyze neural signals to extract information.	Signal capture, feature extraction, and feature clustering.	There are existing commercial applications. Research on massively parallel signal processing and algorithmic design still underway.	The algorithms work on a limited number of signal inputs. In addition, they suffer from high computational complexity and need high computing resources.	*Existing*: Brain–machine interfaces (BMIs)/BCIs, robotic exoskeletons, and prosthetics. *Potential*: Parallel signal processing may dramatically boost the accuracy of the models.

## Data Availability

Not applicable.

## Abbreviations

ADL	Activities of Daily Living
AI	Artificial Intelligence
ANN	Artificial Neural Network
AR	Augmented Reality
BCI	Brain–Computer Interface
CNN	Convolutional Neural Networks
CNS	Central Nervous System
DBS	Deep Brain Stimulation
DC	Direct Current
DL	Deep Learning
DMD	Duchenne Muscular Dystrophy
DOF	Degrees of Freedom
DQN	Deep Q-Networks
ECoG	Electrocorticography
EC-PC	Exponential-Component–Power-Component
EEG	Electroencephalography
EM	Expectation Maximization
EMG	Electromyography
FDA	Food and Drug Administration
FES	Functional Electrical Stimulation
GPU	Graphical Processing Units
HCI	Human–Computer Interaction
HMI	Human–Machine Interface
HRI	Human–Robot Interface
LL	Lower Limb
LSTM	Long Short-Term Memory
MEG	Magnetoencephalography
MEMS	Microelectromechanical Systems
ML	Machine Learning
MRI	Magnetic Resonance Imaging
MUs	Motor Units
NEMS	Nanoelectromechanical Systems
NLU	Natural Language Understanding
OC	Object Classification
PC	Personal Computer
PNS	Peripheral Nervous System
PCA	Principal Component Analysis
RF	Radio Frequency
RL	Reinforcement Learning
RNN	Recurrent Neural Networks
SCI	Spinal Cord Injury
TBI	Traumatic Brain Injury
TDCS	Transcranial Direct Current Stimulation
TMS	Transcranial Magnetic Stimulation
TMSR	Targeted Muscle and Sensory Reinnervation
UL	Upper Limb
VLSI	Very Large-Scale Integration
VR	Virtual Reality
VRE	Virtual Reality Environment
WT	Wavelet Transform
YOLO	You Only Look Once

## References

[1] Gassert R., Dietz V. (2018). Rehabilitation robots for the treatment of sensorimotor deficits: A neurophysiological perspective. J. Neuroeng. Rehabil..

[2] Hobbs B., Artemiadis P. (2020). A Review of Robot-Assisted Lower-Limb Stroke Therapy: Unexplored Paths and Future Directions in Gait Rehabilitation. Front. Neurorobotics.

[3] Kleim J.A., Jones T.A. (2008). Principles of Experience-Dependent Neural Plasticity: Implications for Rehabilitation After Brain Damage. J. Speech Lang. Hear. Res..

[4] Belda-Lois J.M., Horno S.M.-D., Bermejo-Bosch I., Moreno J.C., Pons J.L., Farina D., Iosa M., Molinari M., Tamburella F., Ramos-Murguialday A. (2011). Rehabilitation of gait after stroke: A review towards a top-down approach. J. Neuroeng. Rehabil..

[5] Fasoli S.E., Adans-Dester C.P. (2019). A Paradigm Shift: Rehabilitation Robotics, Cognitive Skills Training, and Function After Stroke. Front. Neurol..

[6] Reinkensmeyer. D.J., Dietz V. (2016). Neurorehabilitation Technology.

[7] Nizamis K., Stienen A.H., Kamper D.G., Keller T., Plettenburg D.H., Rouse E.J., Farina D., Koopman B.F.J.M., Sartori M. (2019). Transferrable Expertise From Bionic Arms to Robotic Exoskeletons: Perspectives for Stroke and Duchenne Muscular Dystrophy. IEEE Trans. Med. Robot. Bionics.

[8] Eapen B.C., Murphy D.P., Cifu D.X. (2017). Neuroprosthetics in amputee and brain injury rehabilitation. Exp. Neurol..

[9] Graimann B., Dietl H. (2013). Introduction to Upper Limb Prosthetics. In Introduction to Neural Engineering for Motor Rehabilitation.

[10] Basteris A., Nijenhuis S.M., Stienen A.H.A., Buurke J.H., Prange G.B., Amirabdollahian F. (2014). Training modalities in robot-mediated upper limb rehabilitation in stroke: A framework for classification based on a systematic review. J. Neuroeng. Rehabil..

[11] Morone G., Masiero S., Werner C., Paolucci S. (2014). Advances in Neuromotor Stroke Rehabilitation. BioMed Res. Int..

[12] Mubin O., Alnajjar F., Jishtu N., Alsinglawi B., Al Mahmud A. (2019). Exoskeletons with Virtual Reality, Augmented Reality, and Gamification for Stroke Patients’ Rehabilitation: Systematic Review. JMIR Rehabil. Assist. Technol..

[13] Fong J., Ocampo R., Tavakoli M. (2020). Intelligent Robotics and Immersive Displays for Enhancing Haptic Interaction in Physical Rehabilitation Environments. Haptic Interfaces for Accessibility, Health, and Enhanced Quality of Life.

[14] Jayaraman A., Marinov B., Singh Y., Burt S., Rymer W.Z. (2020). Current Evidence for Use of Robotic Exoskeletons in Rehabilitation. Wearable Robotics.

[15] Weber L.M., Stein J. (2018). The use of robots in stroke rehabilitation: A narrative review. Neurorehabilitation.

[16] Hidler J., Sainburg R. (2011). Role of Robotics in Neurorehabilitation. Top. Spinal Cord Inj. Rehabil..

[17] Major Z.Z., Vaida C., Major K.A., Tucan P., Simori G., Banica A., Brusturean E., Burz A., Craciunas R., Ulinici I. (2020). The Impact of Robotic Rehabilitation on the Motor System in Neurological Diseases. A Multimodal Neurophysiological Approach. Int. J. Environ. Res. Public Health.

[18] Iandolo R., Marini F., Semprini M., Laffranchi M., Mugnosso M., Cherif A., De Michieli L., Chiappalone M., Zenzeri J. (2019). Perspectives and Challenges in Robotic Neurorehabilitation. Appl. Sci..

[19] Reinkensmeyer D.J., Blackstone S., Bodine C., Brabyn J., Brienza D., Caves K., DeRuyter F., Durfee E., Fatone S., Fernie G. (2017). How a diverse research ecosystem has generated new rehabilitation technologies: Review of NIDILRR’s Rehabilitation Engineering Research Centers. J. Neuroeng. Rehabil..

[20] Poli P., Morone G., Rosati G., Masiero S. (2013). Robotic Technologies and Rehabilitation: New Tools for Stroke Patients’ Therapy. BioMed Res. Int..

[21] Virani S.S., Alonso A., Benjamin E.J., Bittencourt M.S., Callaway C.W., Carson A.P., Chamberlain A.M., Chang A.R., Cheng S., Delling F.N. (2020). Heart Disease and Stroke Statistics—2020 Update: A Report from the American Heart Association. Circulation.

[22] Thrift A.G., Thayabaranathan T., Howard G., Howard V.J., Rothwell P.M., Feigin V.L., Norrving B., Donnan G.A., Cadilhac D.A. (2017). Global stroke statistics. Int. J. Stroke.

[23] Coleman E.R., Moudgal R., Lang K., Hyacinth H.I., Awosika O.O., Kissela B.M., Feng W. (2017). Early Rehabilitation After Stroke: A Narrative Review. Curr. Atheroscler. Rep..

[24] Mazzoleni S., Duret C., Grosmaire A.G., Battini E. (2017). Combining Upper Limb Robotic Rehabilitation with Other Therapeutic Approaches after Stroke: Current Status, Rationale, and Challenges. BioMed Res. Int..

[25] Veerbeek J.M., Langbroek-Amersfoort A.C., Van Wegen E.E.H., Meskers C.G.M., Kwakkel G. (2016). Effects of Robot-Assisted Therapy for the Upper Limb After Stroke. Neurorehabilit. Neural Repair.

[26] Whitfield P.C., Thomas E.O., Summers F., Whyte M., Hutchinson P.J. (2009). Head Injury: A Multidisciplinary Approach.

[27] Dewan M.C., Rattani A., Gupta S., Baticulon R.E., Hung Y.-C., Punchak M., Agrawal A., Adeleye A.O., Shrime M.G., Rubiano A.M. (2019). Estimating the global incidence of traumatic brain injury. J. Neurosurg..

[28] Bigler E.D. (2007). Anterior and middle cranial fossa in traumatic brain injury: Relevant neuroanatomy and neuropathology in the study of neuropsychological outcome. Neuropsychology.

[29] Miotto E.C., Cinalli F.Z., Serrao V.T., Benute G.G., Lucia M.C.S., Scaff M. (2010). Cognitive deficits in patients with mild to moderate traumatic brain injury. Arq. Neuro-Psiquiatria.

[30] Zelek V. (2008). QEEG Brainwave Amplitude and Coherence Values as Predictors of Cognitive Improvement to Neurofeedback After Moderate-to-Severe Acquired Brain Injury. J. Head Trauma Rehabil..

[31] Mathias J.L., Wheaton P. (2007). Changes in attention and information-processing speed following severe traumatic brain injury: A meta-analytic review. Neuropsychology.

[32] Haneef Z., Levin H.S., Frost J.D., Mizrahi E.M. (2013). Electroencephalography and Quantitative Electroencephalography in Mild Traumatic Brain Injury. J. Neurotrauma.

[33] Hoffman D., Stockdale S., Van Egren L. (1996). EEG neurofeedback in the treatment of mild traumatic brain injury. Clin. Electroencephalogr..

[34] Walker J.E., Norman C.A., Weber R.K. (2002). Impact of qEEG-Guided Coherence Training for Patients with a Mild Closed Head Injury. J. Neurother..

[35] Zorcec T., Demerdzieva A., Jordanova P., Qeeg N. (2011). Brain Rate, Executive Functions and Neurofeedback Training in Patients with Traumatic Brain Injury. Acta Inform. Medica.

[36] Barco A., Albo-Canals J., Ng M.K., Garriga C., Callejón L., Turon M., Gomez C., López-Sala A. A robotic therapy for children with TBI. Proceedings of the 2013 8th ACM/IEEE International Conference on Human-Robot Interaction (HRI).

[37] Matarić M., Tapus A., Winstein C., Eriksson J. (2009). Socially assistive robotics for stroke and mild TBI rehabilitation. Stud. Health Technol. Inform..

[38] Sacco K., Cauda F., D’Agata F., Duca S., Zettin M., Virgilio R., Nascimbeni A., Belforte G., Eula G., Gastaldi L. (2011). A combined robotic and cognitive training for locomotor rehabilitation: Evidences of cerebral functional reorganization in two chronic traumatic brain injured patients. Front. Hum. Neurosci..

[39] Debert C.T., Herter T.M., Scott S.H., Dukelow S. (2012). Robotic Assessment of Sensorimotor Deficits After Traumatic Brain Injury. J. Neurol. Phys. Ther..

[40] Nolan K.J., Karunakaran K.K., Ehrenberg N., Kesten A.G. Robotic Exoskeleton Gait Training for Inpatient Rehabilitation in a Young Adult with Traumatic Brain Injury. Proceedings of the 2018 40th Annual International Conference of the IEEE Engineering in Medicine and Biology Society (EMBC).

[41] Resquín F., Gonzalez-Vargas J., Ibáñez J., Brunetti F., Dimbwadyo I., Carrasco L., Alves S., Gonzalez-Alted C., Gomez-Blanco A., Pons J.L. (2017). Adaptive hybrid robotic system for rehabilitation of reaching movement after a brain injury: A usability study. J. Neuroeng. Rehabil..

[42] Antoniou P.E., Athanasiou A., Bamidis P.D. (2020). Virtual and augmented reality in neuroscience. Neurotechnology: Methods, Advances and Applications.

[43] Sessoms P.H., Gottshall K.R., Collins J.-D., Markham A.E., Service K.A., Reini S.A. (2015). Improvements in Gait Speed and Weight Shift of Persons with Traumatic Brain Injury and Vestibular Dysfunction Using a Virtual Reality Computer-Assisted Rehabilitation Environment. Mil. Med..

[44] Zanier E.R., Zoerle T., Di Lernia D., Riva G. (2018). Virtual Reality for Traumatic Brain Injury. Front. Neurol..

[45] Maggio M.G., Torrisi M., Buda A., De Luca R., Piazzitta D., Cannavò A., Leo A., Milardi D., Manuli A., Calabro R.S. (2019). Effects of robotic neurorehabilitation through lokomat plus virtual reality on cognitive function in patients with traumatic brain injury: A retrospective case-control study. Int. J. Neurosci..

[46] 2016 Annual Report - Complete Public Version.pdf. https://www.nscisc.uab.edu/Public/2016%20Annual%20Report%20-%20Complete%20Public%20Version.pdf.

[47] Sweis R., Biller J. (2017). Systemic Complications of Spinal Cord Injury. Curr. Neurol. Neurosci. Rep..

[48] Gowinnage S.S., Arambepola C. (2020). Quality of life and its determinants among community re-integrated soldiers with permanent disabilities following traumatic limb injuries. Qual. Life Res..

[49] Chang F.-H., Liu C.-H., Hung H.-P. (2017). An in-depth understanding of the impact of the environment on participation among people with spinal cord injury. Disabil. Rehabil..

[50] McDaid D., Park A.-L., Gall A., Purcell M., Bacon M. (2019). Understanding and modelling the economic impact of spinal cord injuries in the United Kingdom. Spinal Cord.

[51] Scivoletto G. (2017). The Rehabilitation of Spinal Cord Injury Patients in Europe. Trends Reconstr. Neurosurg..

[52] Mekki M., Delgado A.D., Fry A., Putrino D., Huang V. (2018). Robotic Rehabilitation and Spinal Cord Injury: A Narrative Review. Neurother.

[53] Esquenazi A., Talaty M., Packel A., Saulino M. (2012). The ReWalk Powered Exoskeleton to Restore Ambulatory Function to Individuals with Thoracic-Level Motor-Complete Spinal Cord Injury. Am. J. Phys. Med. Rehabil..

[54] Mehrholz J., A Harvey L., Thomas S., Elsner B. (2017). Is body-weight-supported treadmill training or robotic-assisted gait training superior to overground gait training and other forms of physiotherapy in people with spinal cord injury? A systematic review. Spinal Cord.

[55] Swinnen E., Duerinck S., Baeyens J.-P., Meeusen R., Kerckhofs E. (2010). Effectiveness of robot-assisted gait training in persons with spinal cord injury: A systematic review. J. Rehabil. Med..

[56] Athanasiou A., Arfaras G., Pandria N., Xygonakis I., Foroglou N., Astaras A., Bamidis P.D. (2017). Wireless Brain-Robot Interface: User Perception and Performance Assessment of Spinal Cord Injury Patients. Wirel. Commun. Mob. Comput..

[57] Fakhoury M. (2015). Spinal cord injury: Overview of experimental approaches used to restore locomotor activity. Rev. Neurosci..

[58] Donati A.R.C., Shokur S., Morya E., Campos D.S.F., Moioli R.C., Gitti C.M., Augusto P.B., Tripodi S., Pires C.G., Pereira G.A. (2016). Long-Term Training with a Brain-Machine Interface-Based Gait Protocol Induces Partial Neurological Recovery in Paraplegic Patients. Sci. Rep..

[59] Athanasiou A., Klados M.A., Pandria N., Foroglou N., Kavazidi K.R., Polyzoidis K., Bamidis P.D. (2017). A Systematic Review of Investigations into Functional Brain Connectivity Following Spinal Cord Injury. Front. Hum. Neurosci..

[60] Ahuja C.S., Nori S., Tetreault L., Wilson J., Kwon B., Harrop J., Choi D., Fehlings M.G. (2017). Traumatic Spinal Cord Injury—Repair and Regeneration. Neurosurgery.

[61] Koffler J., Zhu W., Qu X., Platoshyn O., Dulin J.N., Brock J., Graham L., Lu P., Sakamoto J., Marsala M. (2019). Biomimetic 3D-printed scaffolds for spinal cord injury repair. Nat. Med..

[62] Bunday K.L., Urbin M., Perez M.A. (2018). Potentiating paired corticospinal-motoneuronal plasticity after spinal cord injury. Brain Stimul..

[63] Formento E., Minassian K., Wagner F., Mignardot J.B., Le Goff-Mignardot C.G., Rowald A., Bloch J., Micera S., Capogrosso M., Courtine G. (2018). Electrical spinal cord stimulation must preserve proprioception to enable locomotion in humans with spinal cord injury. Nat. Neurosci..

[64] Wagner F.B., Mignardot J.-B., Le Goff-Mignardot C.G., Demesmaeker R., Komi S., Capogrosso M., Rowald A., Seáñez I., Caban M., Pirondini E. (2018). Targeted neurotechnology restores walking in humans with spinal cord injury. Nat. Cell Biol..

[65] Shokur S., Donati A.R.C., Campos D.S.F., Gitti C., Bao G., Fischer D., Almeida S., Braga V.A.S., Augusto P., Petty C. (2018). Training with brain-machine interfaces, visuo-tactile feedback and assisted locomotion improves sensorimotor, visceral, and psychological signs in chronic paraplegic patients. PLoS ONE.

[66] Selfslagh A., Shokur S., Campos D.S.F., Donati A.R.C., Almeida S., Yamauti S.Y., Coelho D.B., Bouri M., Nicolelis M.A.L. (2019). Non-invasive, Brain-controlled Functional Electrical Stimulation for Locomotion Rehabilitation in Individuals with Paraplegia. Sci. Rep..

[67] Cappello L., Meyer J.T., Galloway K.C., Peisner J.D., Granberry R., Wagner D.A., Engelhardt S., Paganoni S., Walsh C.J. (2018). Assisting hand function after spinal cord injury with a fabric-based soft robotic glove. J. Neuroeng. Rehabil..

[68] Mitsopoulos K. (2020). Design of a Prehensile Rehabilitation Assistant for Directed Actuation,” Aristotle University of Thessaloniki, Thessaloniki. http://ikee.lib.auth.gr/record/318391?ln=en.

[69] Ziegler-Graham K., MacKenzie E.J., Ephraim P.L., Travison T.G., Brookmeyer R. (2008). Estimating the Prevalence of Limb Loss in the United States: 2005 to 2050. Arch. Phys. Med. Rehabil..

[70] Wheaton L.A. (2017). Neurorehabilitation in upper limb amputation: Understanding how neurophysiological changes can affect functional rehabilitation. J. Neuroeng. Rehabil..

[71] Sansam K., Neumann V., O’Connor R., Bhakta B. (2009). Predicting walking ability following lower limb amputation: A systematic review of the literature. J. Rehabil. Med..

[72] Esquenazi A. (2004). Amputation rehabilitation and prosthetic restoration. From surgery to community reintegration. Disabil. Rehabil..

[73] Carey S.L., Lura D.J., Highsmith M.J. (2015). Differences in myoelectric and body-powered upper-limb prostheses: Systematic literature review. J. Rehabil. Res. Dev..

[74] Nizamis K., Rijken N.H.M., Van Middelaar R., Neto J., Koopman B.F.J.M., Sartori M. (2020). Characterization of Forearm Muscle Activation in Duchenne Muscular Dystrophy via High-Density Electromyography: A Case Study on the Implications for Myoelectric Control. Front. Neurol..

[75] Pangalila R.F., Bartels B., Bergen M.P., Cobben N.A.M., Stam H.J., Roebroeck M.E. (2011). Upper limb function in adults with Duchenne muscular dystrophy. J. Rehabil. Med..

[76] Darras B.T., Urion D.K., Ghosh P.S., Adam M.P., Ardinger H.H., Pagon R.A., Wallace S.E., Bean L.J., Stephens K., Amemiya A. (1993). Dystrophinopathies. GeneReviews®.

[77] Opstal S.L.S.H., Jansen M., Van Alfen N., De Groot I.J.M. (2013). Health-Related Quality of Life and Its Relation to Disease Severity in Boys with Duchenne Muscular Dystrophy. J. Child Neurol..

[78] Eagle M., Baudouin S.V., Chandler C., Giddings D.R., Bullock R., Bushby K. (2002). Survival in Duchenne muscular dystrophy: Improvements in life expectancy since 1967 and the impact of home nocturnal ventilation. Neuromuscul. Disord..

[79] Bergsma A., Lobo-Prat J., Vroom E., Furlong P., Herder J.L., Corrigan M., De Groot I., Faisal A., Goemans N., Han J. (2016). 1st Workshop on Upper-Extremity Assistive Technology for People with Duchenne: State of the art, emerging avenues, and challenges. Neuromuscul. Disord..

[80] Wagner M.B., Vignos P.J., Carlozzi C. (1989). Duchenne muscular dystrophy: A study of wrist and hand function. Muscle Nerve.

[81] Janssen M.M.H.P., Bergsma A., Geurts A.C.H., De Groot I.J.M. (2014). Patterns of decline in upper limb function of boys and men with DMD: An international survey. J. Neurol..

[82] Pratt J.L. (2016). Control Interfaces to Actively Support the Arm Function of Men with Duchenn Muscular Dystrophy. Ph.D. Thesis.

[83] Mahmood M.N., Peeters L.H.C., Paalman M., Verkerke G.J., Kingma I., Van Dieën J.H. (2018). Development and evaluation of a passive trunk support system for Duchenne muscular dystrophy patients. J. Neuroeng. Rehabil..

[84] Verros S., Mahmood N., Peeters L., Lobo-Prat J., Bergsma A., Hekman E., Verkerke G.J., Koopman B. (2018). Evaluation of Control Interfaces for Active Trunk Support. IEEE Trans. Neural Syst. Rehabil. Eng..

[85] Bos R.A., Nizamis K., Koopman B.F.J.M., Herder J.L., Sartori M., Plettenburg D.H. (2019). A Case Study With Symbihand: An sEMG-Controlled Electrohydraulic Hand Orthosis for Individuals With Duchenne Muscular Dystrophy. IEEE Trans. Neural Syst. Rehabil. Eng..

[86] Bos R.A., Nizamis K., Plettenburg D.H., Herder J.L. Design of an Electrohydraulic Hand Orthosis for People with Duchenne Muscular Dystrophy Using Commercially Available Components. Proceedings of the 2018 7th IEEE International Conference on Biomedical Robotics and Biomechatronics (Biorob).

[87] Nizamis K. (2019). Hand Neuro-Motor Characterization and Motor Intention Decoding in Duchenne Muscular Dystrophy. Ph.D. Thesis.

[88] Desguerre I., Christov C., Mayer M., Zeller R., Becane H.-M., Bastuji-Garin S., Leturcq F., Chiron C., Chelly J., Gherardi R.K. (2009). Clinical Heterogeneity of Duchenne Muscular Dystrophy (DMD): Definition of Sub-Phenotypes and Predictive Criteria by Long-Term Follow-Up. PLoS ONE.

[89] Bushby K., Finkel R., Birnkrant D.J., Case L.E., Clemens P.R., Cripe L., Kaul A., Kinnett K., McDonald C., Pandya S. (2010). Diagnosis and management of Duchenne muscular dystrophy, part 1: Diagnosis, and pharmacological and psychosocial management. Lancet Neurol..

[90] Bushby K., Finkel R., Birnkrant D.J., Case L.E., Clemens P.R., Cripe L., Kaul A., Kinnett K., McDonald C., Pandya S. (2010). Diagnosis and management of Duchenne muscular dystrophy, part 2: Implementation of multidisciplinary care. Lancet Neurol..

[91] Riek L.D. (2016). Robotics Technology in Mental Health Care. Artificial Intelligence in Behavioral and Mental Health Care.

[92] Kleih S., Nijboer F., Halder S., Kübler A. (2010). Motivation modulates the P300 amplitude during brain–computer interface use. Clin. Neurophysiol..

[93] Wood G., Kober S.E., Witte M., Neuper C. (2014). On the need to better specify the concept in brain-computer-interfaces/neurofeedback research. Front. Syst. Neurosci..

[94] Ziemke T., Lowe R., Morse A. (2010). Affective robotics modelling emotion and motivation. Connect. Sci..

[95] Luneski A., Konstantinidis E., Bamidis P.D. (2010). Affective Medicine. Methods Inf. Med..

[96] Rouaix N., Retru-Chavastel L., Rigaud A.-S., Monnet C., Lenoir H., Pino M. (2017). Affective and Engagement Issues in the Conception and Assessment of a Robot-Assisted Psychomotor Therapy for Persons with Dementia. Front. Psychol..

[97] Scoglio A.A., Reilly E.D., Gorman J.A., Drebing C.E. (2019). Use of Social Robots in Mental Health and Well-Being Research: Systematic Review. J. Med. Internet Res..

[98] Jerčić P., Hagelbäck J., Lindley C. (2019). An affective serious game for collaboration between humans and robots. Entertain. Comput..

[99] Lorenzetti V., Melo B., Basílio R., Suo C., Yücel M., Tierra-Criollo C.J., Moll J. (2018). Emotion Regulation Using Virtual Environments and Real-Time fMRI Neurofeedback. Front. Neurol..

[100] Fernández-Caballero A., Navarro E., Fernández-Sotos P., González P., Ricarte J.J., Latorre J.M., Rodriguez-Jimenez R. (2017). Human-Avatar Symbiosis for the Treatment of Auditory Verbal Hallucinations in Schizophrenia through Virtual/Augmented Reality and Brain-Computer Interfaces. Front. Aging Neurosci..

[101] Russell C., Roche A.D., Chakrabarty S. (2019). Peripheral nerve bionic interface: A review of electrodes. Int. J. Intell. Robot. Appl..

[102] Yildiz K.A., Shin A.Y., Kaufman K.R. (2020). Interfaces with the peripheral nervous system for the control of a neuroprosthetic limb: A review. J. Neuroeng. Rehabil..

[103] Cheung K.C. (2007). Implantable microscale neural interfaces. Biomed. Microdevices.

[104] Lazarou I., Nikolopoulos S., Petrantonakis P.C., Kompatsiaris I., Tsolaki M. (2018). EEG-Based Brain–Computer Interfaces for Communication and Rehabilitation of People with Motor Impairment: A Novel Approach of the 21st Century. Front. Hum. Neurosci..

[105] Segato A., Pieri V., Favaro A., Riva M., Falini A., De Momi E., Castellano A. (2019). Automated Steerable Path Planning for Deep Brain Stimulation Safeguarding Fiber Tracts and Deep Gray Matter Nuclei. Front. Robot. AI.

[106] Hong G., Lieber C.M. (2019). Novel electrode technologies for neural recordings. Nat. Rev. Neurosci..

[107] Schwartz A.B. (2016). Movement: How the Brain Communicates with the World. Cell.

[108] Farina D., Negro F. (2012). Accessing the Neural Drive to Muscle and Translation to Neurorehabilitation Technologies. IEEE Rev. Biomed. Eng..

[109] De Luca C.J., Adam A., Wotiz R., Gilmore L.D., Nawab S.H. (2006). Decomposition of Surface EMG Signals. J. Neurophysiol..

[110] Merletti R., Parker P.J. (2004). Electromyography: Physiology, Engineering, and Non-Invasive Applications.

[111] Herrel A., Schaerlaeken V., Ross C., Meyers J., Nishikawa K., Abdala V., Manzano A., Aerts P. (2008). Electromyography and the evolution of motor control: Limitations and insights. Integr. Comp. Biol..

[112] MyoPro Elbow-Wrist-Hand Orthosis (EWHO). https://myomo.com/what-is-a-myopro-orthosis/.

[113] SaeboGlove | Finger Extension Rehabilitation Glove for Stroke Survivors. https://www.saebo.com/saeboglove/.

[114] Kim G.J., Rivera L., Stein J. (2015). Combined Clinic-Home Approach for Upper Limb Robotic Therapy After Stroke: A Pilot Study. Arch. Phys. Med. Rehabil..

[115] Merrill D.R., Lockhart J., Troyk P.R., Weir R.F., Hankin D.L. (2011). Development of an Implantable Myoelectric Sensor for Advanced Prosthesis Control. Artif. Organs.

[116] Heo P., Gu G.M., Lee S.-J., Rhee K., Kim J. (2012). Current hand exoskeleton technologies for rehabilitation and assistive engineering. Int. J. Precis. Eng. Manuf..

[117] Dick S.F., Bert K.U., Bernd L.G., Johannes V.D.P. (2012). High-density Surface EMG: Techniques and Applications at a Motor Unit Level. Biocybern. Biomed. Eng..

[118] Negro F., Orizio C. (2017). Robust estimation of average twitch contraction forces of populations of motor units in humans. J. Electromyogr. Kinesiol..

[119] Farina D., Jiang N., Rehbaum H., Holobar A., Graimann B., Dietl H., Aszmann O.C. (2014). The Extraction of Neural Information from the Surface EMG for the Control of Upper-Limb Prostheses: Emerging Avenues and Challenges. IEEE Trans. Neural Syst. Rehabil. Eng..

[120] Dupan S.S., Krasoulis A., Nazarpour K. Intramuscular EMG For Abstract Myoelectric Control: A Proof Of Concept Study. Proceedings of the 2020 42nd Annual International Conference of the IEEE Engineering in Medicine & Biology Society (EMBC).

[121] Rodrigues C., Fernandeez M., Megia A., Comino N., Del-Ama A., Gil-Agudo A., Jung M.K., Muceli S., Farina D., Moreno J. Comparison of Intramuscular and Surface Electromyography Recordings Towards the Control of Wearable Robots for Incomplete Spinal Cord Injury Rehabilitation. Proceedings of the 2020 8th IEEE RAS/EMBS International Conference for Biomedical Robotics and Biomechatronics (BioRob).

[122] Crouch D.L., Pan L., Filer W., Stallings J.W., Huang H. (2018). Comparing Surface and Intramuscular Electromyography for Simultaneous and Proportional Control Based on a Musculoskeletal Model: A Pilot Study. IEEE Trans. Neural Syst. Rehabil. Eng..

[123] Salminger S., Sturma A., Hofer C., Evangelista M., Perrin M., Bergmeister K.D., Roche A.D., Hasenoehrl T., Dietl H., Farina D. (2019). Long-term implant of intramuscular sensors and nerve transfers for wireless control of robotic arms in above-elbow amputees. Sci. Robot..

[124] Gopura R.A.R.C., Bandara D.S.V., Gunasekara J.M.P. (2013). Recent Trends in EMG-Based Control Methods for Assistive Robots. Electrodiagnosis in New Frontiers of Clinical Research.

[125] Farina D., Vujaklija I., Sartori M., Kapelner T., Negro F., Jiang N., Bergmeister K., Andalib A., Principe J., Aszmann O.C. (2017). Man/machine interface based on the discharge timings of spinal motor neurons after targeted muscle reinnervation. Nat. Biomed. Eng..

[126] Cerone G.L., Botter A., Gazzoni M. (2019). A Modular, Smart, and Wearable System for High Density sEMG Detection. IEEE Trans. Biomed. Eng..

[127] Chowdhury R.H., Reaz M.B.I., Ali M.A.B.M., Bakar A.A.A., Chellappan K., Chang T.G. (2013). Surface Electromyography Signal Processing and Classification Techniques. Sensors.

[128] Hahne J.M., Efarina D., Ejiang N., Eliebetanz D. (2016). A Novel Percutaneous Electrode Implant for Improving Robustness in Advanced Myoelectric Control. Front. Neurosci..

[129] Chadwell A., Kenney L., Thies S., Galpin A., Head J. (2016). The Reality of Myoelectric Prostheses: Understanding What Makes These Devices Difficult for Some Users to Control. Front. Neurorobotics.

[130] Pasquina P.F., Evangelista M., Carvalho A., Lockhart J., Griffin S., Nanos G., McKay P., Hansen M., Ipsen D., Vandersea J. (2015). First-in-man demonstration of a fully implanted myoelectric sensors system to control an advanced electromechanical prosthetic hand. J. Neurosci. Methods.

[131] Farina D., Sartori M. (2016). Surface Electromyography for MAN-Machine Interfacing in Rehabilitation Technologies. Surface Electromyography: Physiology, Engineering, and Applications.

[132] Jiang N., Dosen S., Muller K.-R., Farina D. (2012). Myoelectric Control of Artificial Limbs—Is There a Need to Change Focus? [In the Spotlight]. IEEE Signal Process. Mag..

[133] Durandau G., Farina D., Sartori M. (2017). Robust Real-Time Musculoskeletal Modeling Driven by Electromyograms. IEEE Trans. Biomed. Eng..

[134] Shih J.J., Krusienski D.J., Wolpaw J.R. (2012). Brain-Computer Interfaces in Medicine. Mayo Clin. Proc..

[135] Lotte F., Bougrain L., Cichocki A., Clerc M., Congedo M., Rakotomamonjy A., Yger F. (2018). A review of classification algorithms for EEG-based brain–computer interfaces: A 10 year update. J. Neural Eng..

[136] Nicolas-Alonso L.F., Gomez-Gil J. (2012). Brain Computer Interfaces, a Review. Sensors.

[137] Wang W., Collinger J.L., Perez M.A., Tyler-Kabara E.C., Cohen L.G., Birbaumer N., Brose S.W., Schwartz A.B., Boninger M.L., Weber D.J. (2010). Neural Interface Technology for Rehabilitation: Exploiting and Promoting Neuroplasticity. Phys. Med. Rehabil. Clin. North Am..

[138] Rupp R. (2014). Challenges in clinical applications of brain computer interfaces in individuals with spinal cord injury. Front. Neuroeng..

[139] Athanasiou A., Xygonakis I., Pandria N., Kartsidis P., Arfaras G., Kavazidi K.R., Foroglou N., Astaras A., Bamidis P.D. (2017). Towards Rehabilitation Robotics: Off-the-Shelf BCI Control of Anthropomorphic Robotic Arms. BioMed Res. Int..

[140] Mak J.N., Wolpaw J.R. (2009). Clinical Applications of Brain-Computer Interfaces: Current State and Future Prospects. IEEE Rev. Biomed. Eng..

[141] Al-Quraishi M.S., Elamvazuthi I., Daud S.A., Parasuraman S., Borboni A. (2018). EEG-Based Control for Upper and Lower Limb Exoskeletons and Prostheses: A Systematic Review. Sensors.

[142] Mattia D., Pichiorri F., Molinari M., Rupp R., Allison B.Z., Dunne S., Leeb R., Del R. Millán J., Nijholt A. (2012). Brain Computer Interface for Hand Motor Function Restoration and Rehabilitation. Towards Practical Brain-Computer Interfaces.

[143] Mane R., Chew E., Phua K.S., Ang K.K., Robinson N., Vinod A.P., Guan C. (2019). Prognostic and Monitory EEG-Biomarkers for BCI Upper-Limb Stroke Rehabilitation. IEEE Trans. Neural Syst. Rehabil. Eng..

[144] Dobkin B.H. (2007). Confounders in Rehabilitation Trials of Task-Oriented Training: Lessons From the Designs of the EXCITE and SCILT Multicenter Trials. Neurorehabilit. Neural Repair.

[145] Daly J.J., Cheng R., Hrovat K., Rogers J.M., Litinas K., Dohring M.E. Development and Testing of Non-Invasive BCI + FES/Robot System For Use in Motor Re-Learning After Stroke. Proceedings of the 13th Annual Conference of the International Functional Electrical Stimulation Society “From Movement to Mind”.

[146] Soekadar S.R., Birbaumer N., Slutzky M.W., Cohen L.G. (2015). Brain–machine interfaces in neurorehabilitation of stroke. Neurobiol. Dis..

[147] Vourvopoulos A., Pardo O.M., Lefebvre S., Neureither M., Saldana D., Jahng E., Liew S.-L. (2019). Effects of a Brain-Computer Interface with Virtual Reality (VR) Neurofeedback: A Pilot Study in Chronic Stroke Patients. Front. Hum. Neurosci..

[148] Remsik A., Young B., Vermilyea R., Kiekhoefer L., Abrams J., Elmore S.E., Schultz P., Nair V., Edwards D., Williams J. (2016). A review of the progression and future implications of brain-computer interface therapies for restoration of distal upper extremity motor function after stroke. Expert Rev. Med. Devices.

[149] Rouillard J., Duprès A., Cabestaing F., Leclercq S., Bekaert M.-H., Piau C., Vannobel J.-M., Lecocq C. (2015). Hybrid BCI Coupling EEG and EMG for Severe Motor Disabilities. Procedia Manuf..

[150] Krusienski D.J., Grosse-Wentrup M., Galán F., Coyle D., Miller K.J., Forney E., Anderson C.W. (2011). Critical issues in state-of-the-art brain–computer interface signal processing. J. Neural Eng..

[151] Sengupta S., Basak S., Saikia P., Paul S., Tsalavoutis V., Atiah F., Ravi V., Peters A. (2020). A review of deep learning with special emphasis on architectures, applications and recent trends. Knowl. Based Syst..

[152] Sünderhauf N., Brock O., Scheirer W., Hadsell R., Fox D., Leitner J., Upcroft B., Abbeel P., Burgard W., Milford M. (2018). The limits and potentials of deep learning for robotics. Int. J. Robot. Res..

[153] Shen Y., Ferguson P.W., Rosen J., Rosen J., Ferguson P.W. (2020). Chapter 1 - Upper Limb Exoskeleton Systems—Overview. Wearable Robotics.

[154] Mehrholz J., Thomas S., Kugler J., Pohl M., Elsner B. (2020). Electromechanical-assisted training for walking after stroke. Cochrane Database Syst. Rev..

[155] Ferris D.P. (2009). The exoskeletons are here. J. Neuroeng. Rehabil..

[156] Lee H., Ferguson P.W., Rosen J., Rosen J., Ferguson P.W. (2020). Chapter 11 - Lower Limb Exoskeleton Systems—Overview. Wearable Robotics.

[157] Farris R.J., Quintero H.A., Goldfarb M. Performance evaluation of a lower limb exoskeleton for stair ascent and descent with Paraplegia. Proceedings of the 2012 Annual International Conference of the IEEE Engineering in Medicine and Biology Society.

[158] Wang S.S., Wang L.L., Meijneke C.C., Van Asseldonk E.E., Hoellinger T., Cheron G., Ivanenko Y.Y., La Scaleia V.V., Sylos-Labini F., Molinari M.M. (2015). Design and Control of the MINDWALKER Exoskeleton. IEEE Trans. Neural Syst. Rehabil. Eng..

[159] Hogan N., Krebs H., Charnnarong J., Srikrishna P., Sharon A. MIT-MANUS: A workstation for manual therapy and training. I. Proceedings of the IEEE International Workshop on Robot and Human Communication.

[160] Maciejasz P., Eschweiler J., Gerlach-Hahn K., Jansen-Troy A., Leonhardt S. (2014). A survey on robotic devices for upper limb rehabilitation. J. Neuroeng. Rehabil..

[161] Perry B.E., Evans E.K., Stokic D.S. (2017). Weight compensation characteristics of Armeo®Spring exoskeleton: Implications for clinical practice and research. J. Neuroeng. Rehabil..

[162] Frisoli A., Bergamasco M., Carboncini M.C., Rossi B. (2009). Robotic assisted rehabilitation in Virtual Reality with the L-EXOS. Stud. Heal. Technol. Inform..

[163] Kumar S., Wöhrle H., Trampler M., Simnofske M., Peters H., Mallwitz M., Kirchner E.A., Kirchner F. (2019). Modular Design and Decentralized Control of the Recupera Exoskeleton for Stroke Rehabilitation. Appl. Sci..

[164] Xiloyannis M., Chiaradia D., Frisoli A., Masia L. (2019). Physiological and kinematic effects of a soft exosuit on arm movements. J. Neuroeng. Rehabil..

[165] Riener R., Lünenburger L., Jezernik S., Anderschitz M., Colombo G., Dietz V. (2005). Patient-Cooperative Strategies for Robot-Aided Treadmill Training: First Experimental Results. IEEE Trans. Neural Syst. Rehabil. Eng..

[166] Dimitrousis C., Almpani S., Stefaneas P., Veneman J., Nizamis K., Astaras A. (2020). Neurorobotics: Review of underlying technologies, current developments, and future directions. Neurotechnology Methods Adv. Appl..

[167] Wright J., Macefield V.G., Van Schaik A., Tapson J.C. (2016). A Review of Control Strategies in Closed-Loop Neuroprosthetic Systems. Front. Neurosci..

[168] Carmena J.M. (2013). Advances in Neuroprosthetic Learning and Control. PLoS Biol..

[169] Bumbaširević M., Lesic A., Palibrk T., Milovanovic D., Zoka M., Kravić-Stevović T., Raspopovic S. (2020). The current state of bionic limbs from the surgeon’s viewpoint. EFORT Open Rev..

[170] Morris S., Hirata M., Sugata H., Goto T., Matsushita K., Yanagisawa T., Saitoh Y., Kishima H., Yoshimine T. (2014). Patient-Specific Cortical Electrodes for Sulcal and Gyral Implantation. IEEE Trans. Biomed. Eng..

[171] Serino A., Akselrod M., Salomon R., Martuzzi R., Blefari M.L., Canzoneri E., Rognini G., Van Der Zwaag W., Iakova M., Luthi F. (2017). Upper limb cortical maps in amputees with targeted muscle and sensory reinnervation. Brain.

[172] Kuiken T.A., Barlow A.K., Hargrove L.J., Dumanian G.A. (2017). Targeted Muscle Reinnervation for the Upper and Lower Extremity. Tech. Orthop..

[173] Hargrove L.J., Simon A.M., Young A.J., Lipschutz R.D., Finucane S.B., Smith D.G., Kuiken T.A. (2013). Robotic Leg Control with EMG Decoding in an Amputee with Nerve Transfers. N. Engl. J. Med..

[174] Jönsson S., Caine-Winterberger K., Brånemark R. (2011). Osseointegration amputation prostheses on the upper limbs: Methods, prosthetics and rehabilitation. Prosthet. Orthot. Int..

[175] Malešević N.M., Maneski L.Z.P., Ilić V., Jorgovanović N., Bijelić G., Keller T., Popović D.B. (2012). A multi-pad electrode based functional electrical stimulation system for restoration of grasp. J. Neuroeng. Rehabil..

[176] Popovic-Maneski L.P., Kostic M.D., Bijelic G., Keller T., Mitrovic S., Konstantinovic L., Popovic D.B. (2013). Multi-Pad Electrode for Effective Grasping: Design. IEEE Trans. Neural Syst. Rehabil. Eng..

[177] Hara Y. (2008). Neurorehabilitation with New Functional Electrical Stimulation for Hemiparetic Upper Extremity in Stroke Patients. J. Nippon. Med. Sch..

[178] Müller P., Del Ama A.J., Moreno J.C., Schauer T. (2020). Adaptive multichannel FES neuroprosthesis with learning control and automatic gait assessment. J. Neuroeng. Rehabil..

[179] Franck J.A., Smeets R.J.E.M., Seelen H.A.M. (2018). Evaluation of a functional hand orthosis combined with electrical stimulation adjunct to arm-hand rehabilitation in subacute stroke patients with a severely to moderately affected hand function. Disabil. Rehabil..

[180] Peters T.E., Bhavaraju N.C., Frei M.G., Osorio I. (2001). Network System for Automated Seizure Detection and Contingent Delivery of Therapy. J. Clin. Neurophysiol..

[181] Wilder A., Hiatt S., Dowden B., Brown N., Normann R., Clark G. (2009). Automated Stimulus-Response Mapping of High-Electrode-Count Neural Implants. IEEE Trans. Neural Syst. Rehabil. Eng..

[182] Markovic M., Dosen S., Cipriani C., Popovic D., Farina D. (2014). Stereovision and augmented reality for closed-loop control of grasping in hand prostheses. J. Neural Eng..

[183] Shpigelman L., Lalazar H., Vaadia E., Koller D., Schuurmans D., Bengio Y., Bottou L. (2009). Kernel-ARMA for Hand Tracking and Brain-Machine interfacing During 3D Motor Control. Advances in Neural Information Processing Systems 21.

[184] Li Z., O’Doherty J.E., Hanson T.L., Lebedev M.A., Henriquez C.S., Nicolelis M.A.L. (2009). Unscented Kalman Filter for Brain-Machine Interfaces. PLoS ONE.

[185] DiGiovanna J., Mahmoudi B., Fortes J., Principe J.C., Sanchez J.C. (2009). Coadaptive Brain–Machine Interface via Reinforcement Learning. IEEE Trans. Biomed. Eng..

[186] Aggarwal V., Singhal G., He J., Schieber M.H., Thakor N.V. Towards closed-loop decoding of dexterous hand movements using a virtual integration environment. Proceedings of the 2008 30th Annual International Conference of the IEEE Engineering in Medicine and Biology Society.

[187] Ehrsson H.H., Rosén B., Stockselius A., Ragnö C., Köhler P., Lundborg G. (2008). Upper limb amputees can be induced to experience a rubber hand as their own. Brain.

[188] Manero A., Smith P., Sparkman J., Dombrowski M., Courbin D., Kester A., Womack I., Chi A. (2019). Implementation of 3D Printing Technology in the Field of Prosthetics: Past, Present, and Future. Int. J. Environ. Res. Public Health.

[189] Kate J.T., Smit G., Breedveld P. (2017). 3D-printed upper limb prostheses: A review. Disabil. Rehabil. Assist. Technol..

[190] Windrich M., Grimmer M., Christ O., Rinderknecht S., Beckerle P. (2016). Active lower limb prosthetics: A systematic review of design issues and solutions. Biomed. Eng. Online.

[191] Azocar A.F., Mooney L.M., Duval J.-F., Simon A.M., Hargrove L.J., Rouse E.J. (2020). Design and clinical implementation of an open-source bionic leg. Nat. Biomed. Eng..

[192] VijayaVenkataRaman S., Fuh J.Y.H., Lu W.F. (2017). 3D Printing and 3D Bioprinting in Pediatrics. Bioengeniring.

[193] Burdea G.C., Coiffet P. (2003). Virtual Reality Technology. Presence: Teleoperators Virtual Environ..

[194] Azuma R., Baillot Y., Behringer R., Feiner S., Julier S., MacIntyre B. (2001). Recent advances in augmented reality. IEEE Eng. Med. Boil Mag..

[195] Holden M.K. (2005). Virtual Environments for Motor Rehabilitation: Review. CyberPsychology Behav..

[196] Keshner E.A. (2004). Virtual reality and physical rehabilitation: A new toy or a new research and rehabilitation tool?. J. Neuroeng. Rehabil..

[197] Bohil C.J., Alicea B., Biocca F.A. (2011). Virtual reality in neuroscience research and therapy. Nat. Rev. Neurosci..

[198] Howard M.C. (2017). A meta-analysis and systematic literature review of virtual reality rehabilitation programs. Comput. Hum. Behav..

[199] Sucar L.E., Orihuela-Espina F., Velazquez R.L., Reinkensmeyer D.J., Leder R., Hernandez-Franco J. (2014). Gesture Therapy: An Upper Limb Virtual Reality-Based Motor Rehabilitation Platform. IEEE Trans. Neural Syst. Rehabil. Eng..

[200] Huang H., Wolf S.L., He J. (2006). Recent developments in biofeedback for neuromotor rehabilitation. J. Neuroeng. Rehabil..

[201] Verma R., Arya K.N., Garg R., Singh T. (2011). Task-Oriented Circuit Class Training Program with Motor Imagery for Gait Rehabilitation in Poststroke Patients: A Randomized Controlled Trial. Top. Stroke Rehabil..

[202] Chen Y., Huang H., Xu W., Wallis R.I., Sundaram H., Rikakis T., Ingalls T., Olson L., He J. The design of a real-time, multimodal biofeedback system for stroke patient rehabilitation. Proceedings of the 14th ACM International Conference on Multimedia.

[203] Michalski S.C., Szpak A., Saredakis D., Ross T.J., Billinghurst M., Loetscher T. (2019). Getting your game on: Using virtual reality to improve real table tennis skills. PLoS ONE.

[204] De Araújo A.V.L., Neiva J.F.D.O., Monteiro C.B.D.M., Magalhães F.H. (2019). Efficacy of Virtual Reality Rehabilitation after Spinal Cord Injury: A Systematic Review. BioMed Res. Int..

[205] Tageldeen M.K., Elamvazuthi I., Perumal N., Ganesan T. A virtual reality based serious games for rehabilitation of arm. Proceedings of the 2017 IEEE 3rd International Symposium in Robotics and Manufacturing Automation (ROMA).

[206] Comani S., Velluto L., Schinaia L., Cerroni G., Serio A., Buzzelli S., Sorbi S., Guarnieri B. (2015). Monitoring Neuro-Motor Recovery from Stroke with High-Resolution EEG, Robotics and Virtual Reality: A Proof of Concept. IEEE Trans. Neural Syst. Rehabil. Eng..

[207] Eng K., Siekierka E., Pyk P., Chevrier E., Hauser Y., Cameirao M., Holper L., Hägni K., Zimmerli L., Duff A. (2007). Interactive visuo-motor therapy system for stroke rehabilitation. Med. Biol. Eng. Comput..

[208] Anderson F., Grossman T., Matejka J., Fitzmaurice G. YouMove. Proceedings of the 26th Annual ACM Symposium on User interface Software and Technology.

[209] Hondori H.M., Khademi M., Dodakian L., McKenzie A., Lopes C.V., Cramer S.C. (2016). Choice of Human–Computer Interaction Mode in Stroke Rehabilitation. Neurorehabilit. Neural Repair.

[210] Garrett B., Taverner T., Gromala D., Tao G., Cordingley E., Sun C. (2018). Virtual Reality Clinical Research: Promises and Challenges. JMIR Serious Games.

[211] Slater M., Wilbur S. (1997). A framework for immersive virtual environments (FIVE): Speculations on the role of presence in virtual environments. Presence.

[212] Slater M., Lotto B., Arnold M.M., Sanchez-Vives M.V. (2009). How we experience immersive virtual environments: The concept of presence and its measurement. Anu. Psicol..

[213] Steuer J. (1992). Defining Virtual Reality: Dimensions Determining Telepresence. J. Commun..

[214] Weech S., Kenny S., Barnett-Cowan M. (2019). Presence and Cybersickness in Virtual Reality Are Negatively Related: A Review. Front. Psychol..

[215] Raaen K., Kjellmo I., Chorianopoulos K., Divitini M., Baalsrud Hauge J., Jaccheri L., Malaka R. (2015). Measuring Latency in Virtual Reality Systems. Proceedings of the Entertainment Computing - ICEC 2015.

[216] Oña E.D., Garcia-Haro J.M., Jardón A., Balaguer C. (2019). Robotics in Health Care: Perspectives of Robot-Aided Interventions in Clinical Practice for Rehabilitation of Upper Limbs. Appl. Sci..

[217] Sim D.Y.Y., Loo C.K. (2015). Extensive assessment and evaluation methodologies on assistive social robots for modelling human–robot interaction – A review. Inf. Sci..

[218] WHO|Neurological Disorders: Public Health Challenges,” WHO. https://www.who.int/mental_health/neurology/neurodiso/en/.

[219] Karikari T.K., Charway-Felli A., Höglund K., Blennow K., Zetterberg H. (2018). Commentary: Global, regional, and national burden of neurological disorders during 1990–2015: A systematic analysis for the Global Burden of Disease Study 2015. Front. Neurol..

[220] Cassimatis N.L., Trafton J.G., Bugajska M.D., Schultz A.C. (2004). Integrating cognition, perception and action through mental simulation in robots. Robot. Auton. Syst..

[221] Laird J.E., Lebiere C., Rosenbloom P.S. (2017). A Standard Model of the Mind: Toward a Common Computational Framework across Artificial Intelligence, Cognitive Science, Neuroscience, and Robotics. AI Mag..

[222] Zhao Z.-Q., Zheng P., Xu S.-T., Wu X. (2019). Object Detection with Deep Learning: A Review. IEEE Trans. Neural Netw. Learn. Syst..

[223] Aoki Y., Goforth H., Srivatsan R.A., Lucey S. PointNetLK: Robust & Efficient Point Cloud Registration Using PointNet. Proceedings of the 2019 IEEE/CVF Conference on Computer Vision and Pattern Recognition (CVPR).

[224] Redmon J., Divvala S., Girshick R., Farhadi A. You Only Look Once: Unified, Real-Time Object Detection. Proceedings of the 2016 IEEE Conference on Computer Vision and Pattern Recognition (CVPR).

[225] Kramer E.R., Sáinz A.O., Mitrevski A., Plöger P.G., Chalup S., Niemueller T., Suthakorn J., Williams M.-A. (2019). Tell Your Robot What to Do: Evaluation of Natural Language Models for Robot Command Processing. Robot World Cup.

[226] Mnih V. (2013). Playing Atari with Deep Reinforcement Learning. ArXiv.

[227] Lillicrap T.P. (2019). Continuous control with deep reinforcement learning. ArXiv.

[228] Schuld M., Sinayskiy I., Petruccione F. (2014). An introduction to quantum machine learning. Contemp. Phys..

[229] Biamonte J., Wittek P., Pancotti N., Rebentrost P., Wiebe N., Lloyd S. (2017). Quantum machine learning. Nat. Cell Biol..

[230] Haufe F.L., Wolf P., Riener R. (2020). Human-in-the-loop optimization of a multi-joint wearable robot for movement assistance. Proc. Autom. Med Eng..

[231] EMERGING FRONTIERS IN RESEARCH AND INNOVATION (EFRI): Distributed Chemical Manufacturing (DCheM) and Engineering the Elimination of End-of-Life Plastics (E3P) | NSF National Science Foundation. https://www.nsf.gov/funding/pgm_summ.jsp?pims_id=13708.

[232] Quiroga R.Q., Nadasdy Z., Ben-Shaul Y. (2004). Unsupervised Spike Detection and Sorting with Wavelets and Superparamagnetic Clustering. Neural Comput..

[233] Bonnet S., Bêche J.-F., Gharbi S., Abdoun O., Bocquelet F., Joucla S., Agache V., Sauter F., Pham P., Dupont F. (2012). NeuroPXI: A real-time multi-electrode array system for recording, processing and stimulation of neural networks and the control of high-resolution neural implants for rehabilitation. IRBM.

[234] Wu T., Yang Z. Power-efficient VLSI implementation of a feature extraction engine for spike sorting in neural recording and signal processing. Proceedings of the 2014 13th International Conference on Control Automation Robotics & Vision (ICARCV).

[235] Chen Z. (2017). A Primer on Neural Signal Processing. IEEE Circuits Syst. Mag..

[236] El-Samie F.E.A., Alotaiby T.N., Khalid M.I., Alshebeili S.A., Aldosari S.A. (2018). A Review of EEG and MEG Epileptic Spike Detection Algorithms. IEEE Access.

[237] Johansen A.R., Jin J., Maszczyk T., Dauwels J., Cash S.S., Westover M.B. Epileptiform spike detection via convolutional neural networks. Proceedings of the 2016 IEEE International Conference on Acoustics, Speech and Signal Processing (ICASSP).

[238] Lewicki M.S. (1998). A review of methods for spike sorting: The detection and classification of neural action potentials. Netw. Comput. Neural Syst..

[239] Kilicarslan A., Prasad S., Grossman R.G., Contreras-Vidal J.L. High accuracy decoding of user intentions using EEG to control a lower-body exoskeleton. Proceedings of the 2013 35th Annual International Conference of the IEEE Engineering in Medicine and Biology Society (EMBC).

[240] Kwon K.Y., Eldawlatly S., Oweiss K. (2012). NeuroQuest: A comprehensive analysis tool for extracellular neural ensemble recordings. J. Neurosci. Methods.

[241] Burns M.K., Pei D., Vinjamuri R. (2019). Myoelectric Control of a Soft Hand Exoskeleton Using Kinematic Synergies. IEEE Trans. Biomed. Circuits Syst..

[242] Tam W.-K., Yang Z. (2018). Neural Parallel Engine: A toolbox for massively parallel neural signal processing. J. Neurosci. Methods.

[243] Chen D., Wang L., Ouyang G., Li X. (2011). Massively Parallel Neural Signal Processing on a Many-Core Platform. Comput. Sci. Eng..

[244] Basic Neuroscience|National Institute of Neurological Disorders and Stroke. https://www.ninds.nih.gov/Current-Research/Research-Funded-NINDS/Basic-Neuroscience.

[245] Turner D.L., Emurguialday A.R., Ebirbaumer N., Ehoffmann U., Eluft A. (2013). Neurophysiology of Robot-Mediated Training and Therapy: A Perspective for Future Use in Clinical Populations. Front. Neurol..

[246] Severini G., Koenig A., Adans-Dester C., Cajigas I., Cheung V.C.K., Bonato P. (2020). Robot-Driven Locomotor Perturbations Reveal Synergy-Mediated, Context-Dependent Feedforward and Feedback Mechanisms of Adaptation. Sci. Rep..

[247] Cajigas I., Koenig A., Severini G., Smith M., Bonato P. (2017). Robot-induced perturbations of human walking reveal a selective generation of motor adaptation. Sci. Robot..

[248] Ebolognini N., Erusso C., Evallar G. (2015). Crossmodal illusions in neurorehabilitation. Front. Behav. Neurosci..

[249] Cumming D.R.S., Furber S.B., Paul D.J. (2014). Beyond Moore’s law. Philos. Trans. R. Soc. A Math. Phys. Eng. Sci..

[250] Chen Z., Li Z., Li J., Liu C., Lao C., Fu Y., Liu C., Li Y., Wang P., He Y. (2019). 3D printing of ceramics: A review. J. Eur. Ceram. Soc..

[251] Frazier W.E. (2014). Metal Additive Manufacturing: A Review. J. Mater. Eng. Perform..

[252] Munaz A., Vadivelu R.K., John J.S., Barton M., Kamble H., Nguyen N.-T. (2016). Three-dimensional printing of biological matters. J. Sci. Adv. Mater. Devices.

[253] Gao B., Yang Q., Zhao X., Jin G., Ma Y., Xu F. (2016). 4D Bioprinting for Biomedical Applications. Trends Biotechnol..

[254] Kaur K., Noor A. (2011). Strategies & Methodologies for Low Power VLSI Designs: A Review. Int. J. Adv. Eng. Technol..

[255] Gul J.Z., Sajid M., Rehman M.M., Siddiqui G.U., Shah I., Kim K.-H., Lee J.-W., Choi K.H. (2018). 3D printing for soft robotics—A review. Sci. Technol. Adv. Mater..

[256] Sojan S., Kulkarni R. (2016). A Comprehensive Review of Energy Harvesting Techniques and its Potential Applications. Int. J. Comput. Appl..

[257] Seo D., Carmena J.M., Rabaey J.M., Alon E., Maharbiz M.M. (2013). Neural Dust: An Ultrasonic, Low Power Solution for Chronic Brain-Machine Interfaces. ArXiv.

[258] Warneke B., Last M., Liebowitz B., Pister K. (2001). Smart Dust: Communicating with a cubic-millimeter computer. Computers.

[259] Zrenner C., Ebelardinelli P., Emüller-Dahlhaus F., Eziemann U. (2016). Closed-Loop Neuroscience and Non-Invasive Brain Stimulation: A Tale of Two Loops. Front. Cell. Neurosci..

[260] Broccard F.D., Mullen T., Chi Y.M., Peterson D., Iversen J.R., Arnold M., Kreutz-Delgado K., Jung T.-P., Makeig S., Poizner H. (2014). Closed-Loop Brain–Machine–Body Interfaces for Noninvasive Rehabilitation of Movement Disorders. Ann. Biomed. Eng..

[261] Stroppa F., Loconsole C., Frisoli A. (2018). Convex polygon fitting in robot-based neurorehabilitation. Appl. Soft Comput..

[262] Wutzl B., Leibnitz K., Rattay F., Kronbichler M., Murata M., Golaszewski S.M. (2019). Genetic algorithms for feature selection when classifying severe chronic disorders of consciousness. PLoS ONE.

[263] Neely R.M., Piech D.K., Santacruz S.R., Maharbiz M.M., Carmena J.M. (2018). Recent advances in neural dust: Towards a neural interface platform. Curr. Opin. Neurobiol..

[264] Seo D., Neely R.M., Shen K., Singhal U., Alon E., Rabaey J.M., Carmena J.M., Maharbiz M.M. (2016). Wireless Recording in the Peripheral Nervous System with Ultrasonic Neural Dust. Neuron.

[265] Reinkensmeyer D.J. (2019). JNER at 15 years: Analysis of the state of neuroengineering and rehabilitation. J. Neuroeng. Rehabil..

[266] Stinear C.M., Lang C.E., Zeiler S., Byblow W.D. (2020). Advances and challenges in stroke rehabilitation. Lancet Neurol..

[267] Musselman K.E., Shah M., Zariffa J. (2018). Rehabilitation technologies and interventions for individuals with spinal cord injury: Translational potential of current trends. J. Neuroeng. Rehabil..

[268] Song W.-K. (2016). Trends in rehabilitation robots and their translational research in National Rehabilitation Center, Korea. Biomed. Eng. Lett..

